# Predicting cognitive decline: Comparative analysis of ANU-ADRI, CAIDE, CogDrisk, LIBRA, LIBRA2, UKBDRS and Lancet based dementia risk scores in the HUNT study

**DOI:** 10.1016/j.tjpad.2026.100524

**Published:** 2026-02-28

**Authors:** Josephine Stubs, Geir Selbæk, Bjørn Heine Strand, Gill Livingston, Kaarin J. Anstey, Kay Deckers, Mika Kivimäki, Steinar Krokstad, Fiona E. Mathews, Ellen Melbye Langballe

**Affiliations:** a Norwegian National Centre for Ageing and Health, Vestfold Hospital Trust, Tønsberg, Aldring og helse, 3103 Tønsberg, Norway; b Department of Geriatric Medicine, Oslo University Hospital, Oslo, Kirkeveien 166, 0450 Oslo, Norway; c Faculty of Medicine, Institute of Clinical Medicine, University of Oslo, Kirkeveien 166, 0450 Oslo, Norway; d Department of Physical Health and Ageing, Norwegian Institute of Public Health, Oslo, Norway; e Division of Psychiatry, 149 Tottenham Court Rd, University College London, London, W1W 7EJ, UK; f North London NHS Foundation Trust, St Pancras Hospital, London, NW1 0PE, UK; g School of Psychology, University of New South Wales, Sydney, Australia; h Neuroscience Research Australia, Sydney, Australia; i UNSW Ageing Futures Institute, University of New South Wales, Sydney, Australia; j Alzheimer Centrum Limburg, Department of Psychiatry and Neuropsychology, Mental Health and Neuroscience Research Institute (MHeNs), Maastricht University, Dr. Tanslaan 12, 6229 ET, Maastricht, the Netherlands; k Department of Public Health, Faculty of Medicine, University of Helsinki, Tukholmankatu 8 B, FI-00014, Finland; l Department of Public Health and Nursing, Faculty of Medicine and Health Sciences, HUNT Research Centre, Norwegian University of Science and Technology, Trondheim, Norway; m Levanger Hospital, Nord-Trøndelag Hospital Trust, Levanger, Norway; n Institute for Clinical and Applied Health Research, University of Hull, Cottingham Road, Hull, HU6 7RX, UK

**Keywords:** Dementia risk index, Modifiable risk factors, HUNT, Lifestyle risk, Aging, Cognitive decline

## Abstract

**Objective:**

To evaluate and compare the predictive value of eight dementia risk scores for late-life cognitive function and cognitive decline; ANU-ADRI, CAIDE, CogDrisk, LIBRA, LIBRA2, UKBDRS(-APOE), and a Lancet commission-based risk score.

**Methods:**

Using Norwegian Trøndelag Health Study (HUNT) data, we calculated risk scores from lifestyle and health data of 7221 dementia-free participants (mean age: 76.8 years, 54.1% female) collected in HUNT3 (2006–2008). Cognitive function was assessed using the Montreal Cognitive Assessment scale (MoCA) 11 years later in HUNT4 70+, and reassessed in 4716 participants 4 years thereafter. Associations between continuous risk scores or risk score tertiles, cognition and cognitive decline were examined using linear mixed-effects models. Logistic regression models were used to test associations between risk scores and *a* ≥ 3-point decline in MoCA scores.

**Results:**

All risk scores were significantly associated with cognitive function and cognitive decline. Associations with cognitive function ranged from UKBDRS β _per 1SD_=-1.61(95%CI:-1.72,-1.51) to CAIDE (β=-0.74;95%CI:-0.82,-0.67), and with yearly cognitive decline from Lancet (β=-0.23;95%CI:-0.27,-0.18) to CAIDE (β=-0.04;95%CI:-0.07,-0.02). High-low risk group differences in cognitive function were largest for CogDrisk (β=-3.04;95%CI:-3.27,-2.81), LIBRA (β=-3.04;95%CI:-3.27,-2.80) and lowest for CAIDE (β=-1.65;95%CI:-1.86,-1.44). High-risk groups showed the steepest decline for UKBDRS-APOE (β=-0.43;95%CI:-0.52,-0.34), Lancet (β=-0.39;95%CI:-0.48,-0.30), and LIBRA (β=-0.38;95%CI:-0.47,-0.28). All scores predicted ≥3-point decline modestly: AUCs were highest for UKBDRS (AUC=0.61;95%CI:0.60,0.63), UKBDRS-APOE (0.61;95%CI:0.60,0.63), CogDrisk (0.60;95%CI:0.58,0.62), and Lancet (0.60;95%CI:0.58,0.61), but none outperformed a model including age and education alone (0.61;95%CI:0.60,0.63).

**Conclusion:**

Risk scores captured meaningful gradients in cognition and decline but offered limited discriminatory accuracy beyond demographics, supporting their use for prevention-oriented risk profiling rather than prediction.

## Highlights

• Eight dementia risk scores and a demographics model were compared longitudinally.
• UKBDRS, LIBRA, ANU-ADRI best predicted cognition 11 years after risk assessment.
• Lancet, UKBDRS, ANU-ADRI best predicted 4-year cognitive decline.
• UKBDRS, CogDrisk, Lancet best predicted who would get significant cognitive decline.
• No risk score outperformed age and education alone in predicting cognitive decline.

## 1 Introduction

Cognitive decline, including gradual reductions in episodic memory, processing speed, and executive function, is part of normal aging and does not necessarily indicate disease development [1,2]. However, pronounced cognitive decline is associated with reduced quality of life [3], greater dependency [4], increased risk of disease, injury, healthcare utilization [4] and progression to dementia [2,5], posing a substantial personal, social, and economic burden [3,6,7].

Growing evidence suggests that the risk and severity of age-related cognitive decline may be influenced by several lifestyle factors, including social, cognitive and physical activity, diet, smoking, sleep, and cardiovascular risk factors [[8], [9], [10]]. These findings have motivated efforts to establish comprehensive overviews of modifiable risk and protective factors for the prevention of cognitive decline and dementia, leading to the development of prevention frameworks and multiple risk indices [11,12]. Some of these indices are available as digital tools, such as apps, web-based self-assessments, and validated questionnaires for use by individuals, clinicians and researchers. Meanwhile, the individual risk factors and risk profiles provide guidelines for global prevention strategies [10].

One of the most influential frameworks that summerizes potentially modifiable risk is the Lancet Commission on Dementia Prevention, Intervention and Care, updated in 2024 [13]. Through systematic reviews and meta-analyses, it identified 14 modifiable risk factors that, if fully addressed, could achieve a potential reduction of 45% of dementia cases [13] (Supplementary Table 1). This framework is widely regarded as the current gold standard and has been incorporated into the World Health Organization’s Global Action Plan on the public health response to dementia [10,14,15]. However, thus far the Lancet framework has primarily been used for the development of prevention strategies and policy guidance. While the Lancet Commission also reports risk ratios appropriate for individual risk assessment, it has not yet been operationalized or externally validated for individual risk prediction or reduction.

In parallel, several dementia risk scores have been developed specifically for individual risk prediction. Each risk score was developed using slightly different approaches to identify, include and weight risk factors which were associated with brain health and dementia risk [[16], [17], [18], [19], [20]] (Supplementary Table 1). The most established and well-validated [11,[21], [22], [23], [24]] examples include the Australian National University Alzheimer’s Disease Risk Index (ANU-ADRI) [17], the Cardiovascular Risk Factors, Aging, and Incidence of Dementia (CAIDE) score [16], the Cognitive Health and Dementia Risk Assessment (CogDrisk) tool [18], the LIfestyle for BRAin Health (LIBRA) index [19] and its updated version LIBRA2 [20]. A recent addition is the UK Biobank Dementia Risk Score (UKBDRS, and its expanded version UKBDRS-APOE including ApoE4 genotyping) [25], which performed well at dementia risk prediction in UK Biobank and Whitehall II studies relative to other dementia risk scores [25].

Initially designed to assess dementia risk and guide prevention at individual or population levels [26], these risk scores are increasingly applied in research to study associations with cognitive decline [26,27] and dementia risk [11]; to examine neural correlates of aging [26]; to support trial participant selection and population stratification [26], competing risk modeling [20], [26]; and as cognitive or behavioral outcomes [28,29] or surrogate endpoints in prevention trials [27,29]. However, only ANU-ADRI and LIBRA have been compared directly for their ability to predict cognitive decline [30]. This is an important limitation, as cognitive decline often represents the earliest clinical manifestation of dementia [2,5], and even without conversion to dementia, is associated with substantial functional, social, and economic burden [3,4]. Moreover, none of the existing risk scores incorporate all the most recent Lancet Commission risks, which itself has not been formalized as a quantitative index.

While each framework has its own strengths (Supplementary Table 1), the proliferation of different indices and risk factors [11,12,31] has made it difficult for clinicians, researchers, and policymakers to determine which, if any, should be prioritized for risk evaluation or methodological use. Despite extensive validation individually, to our knowledge, no comparative research has systematically evaluated the relative performance of these scores for prediction of cognitive function and cognitive decline within the same population.

To address these gaps, we analysed data from the HUNT study, a large, representative cohort of a Norwegian population, compared eight frequently cited risk scores – ANU-ADRI, CAIDE, CogDrisk, LIBRA, LIBRA2, UKBDRS(-APOE), and a Lancet Commission-based risk score designed for this study – in relation to subsequent cognitive function and cognitive decline over 4.2 years. To examine potential demographic differences, we further assessed whether predictive performance varied by age at risk factor measurement. Lastly, we evaluated the ability of these scores to discriminate between individuals who do or do not experience significant cognitive decline.

## 2 Method

### 2.1 Study population

This study used data from the Trøndelag Health Study (HUNT), an ongoing population-based cohort in Norway. Since 1984, HUNT has collected extensive health, lifestyle, and medical information through four main study waves conducted approximately every 10–11 years along with multiple sub-studies [32]. At all waves, all adults in the Nord-Trøndelag area were invited to participate. Linkage to Statistics Norway (SSB) and Norwegian Patient Registry (NPR) provided additional data on education and International Classification of Diseases (ICD)-10 diagnoses from the years 2007–2009. All the included HUNT participants have provided informed consent for participation and linkage to national health registries. The present project was approved by the Regional Committee for Medical and Health Research Ethics (REK Southeast #251687) and the Norwegian Agency for Shared Services in Education and Research (Formerly Norwegian Center for Research Data, (NSD #571736)).

This study draws on three data collection points from the HUNT Study. From the first collection point, the HUNT3 Survey completed in 2006–2008, we gathered information about lifestyle and risk factor information used to establish dementia risk groups. HUNT3 includes nearly all variables required to compute dementia risk scores and coincides with the start of diagnostic coverage in the NPR in 2007. For the second collection point; HUNT4 70+ (2017–2019), all regional individuals aged 70 years and older were invited to undergo cognitive, physical, and psychosocial assessment. This collection point served as the first cognitive assessment. The third collection point; HUNT Ageing in Trøndelag (AiT; 2021–2023) was a 4-year follow-up to HUNT4 70+, reassessing cognitive, physical, and mental health in the same cohort [33]. Repeated cognitive data were used to assess changes in cognitive function.

Of the 9 930 individuals who participated in HUNT4 70+ (representing 51.2 % of the local population aged 70 and older in the region [34]), 8 219 (82.8 %) were dementia free (see Supplementary Table 2 for diagnostic methods), and 7 221 (72.7%) had measurement of risk factors from 2006–2008, and cognitive assessments from 2017–2019. Among them, 4716 (65.3 %) had also data from a cognitive reassessment in 2021–2023.

### 2.2 Cognitive assessments

Cognitive function was assessed by trained personnel in both HUNT4 70+ and AiT using the Montreal Cognitive Assessment (MoCA), a standardized short-form test battery covering memory, executive and visuospatial abilities, attention, language, abstraction, and orientation. Scores range from 0 to 30, with higher scores indicating better cognitive performance. MoCA was first administered during HUNT4 70+, approximately 10.6 years after risk-factor assessment at HUNT3. The second MoCA assessment was 4.2 years later at AiT, corresponding to 14.8 years after HUNT3 [35].

### 2.3 Data preparation and risk score generation

All risk score variables were derived from HUNT3 (2006–2008) and, where HUNT3 data was missing, supplemented with data from earlier waves (HUNT1: 1984–1986; HUNT2: 1995–1997) and external SSB (2007) and NPR (2007–2009) data to minimize missingness. Variables were recoded and harmonized to align with the definitions, cut-offs, and weighting schemes specified by each individual risk algorithm in line with previously published methods [23] (see Supplementary Table 1 and 2). A risk score based on the Lancet Commission risk factors (hereafter referred to as the Lancet-based risk score) was constructed specifically for this study by coding each risk variable identified by the Lancet report as absent (=0) or as present (=1 x risk factor weight) weighting it using the risk ratios identified in the Lancet Commission; weighted values were summed to derive a total score (see Table 1). This approach closely mirrors the methods used to develop the seven other risk scores examined in this study [[16], [17], [18], [19], [20],^25^]. The Lancet Commission report includes only potentially modifiable risk factors and therefore does not include age and sex as a risk factor. Therefore, ANU-ADRI age and sex weights were added to the Lancet risk score to enable head-to-head comparison of all risk scores. The same was done with the LIBRA risk scores, which also do not include non-modifiable dementia risk factors such as age, sex and education. Additionally, all analyses were replicated with age, sex and education weights excluded from all risk scores.

**Table 1:** Descriptives of the HUNT study participants.

Variable	Total sample *N* = 7 221; n (%)	AiT available *n* = 4 716; n (%)	AiT missing *n* = 2 505; n (%)
**Sex**			
Female	3 904 (54.06 %)	2 546 (53.99 %)	1 358 (54.21 %)
Male	3 317 (45.94 %)	2 170 (46.01 %)	1 147 (45.79 %)
**Education; M (SD)**	12.69 (2.25)	12.94 (2.67)	12.20 (2.14)
< 8 y	5 (0.07 %)	1 (0.02 %)	4 (0.16 %)***
8–12 y	4 686 (64.89 %)	2 855 (60.54 %)	1 831 (73.09 %)***
≥ 12 y	2 530 (35.04 %)	1 860 (39.44 %)	670 (26.75 %)***
**Age group**			
< 65 y	3 563 (49.64 %)	2 600 (55.46 %)	963 (38.69 %)***
≥ 65 y	3 614 (50.36 %)	2 088 (44.54 %)	1 526 (61.31 %)***
	**M (SD) [Min-Max]**	**M (SD) [Min-Max]**	**M (SD) [Min-Max]**
Age (HUNT3)	66.31 (5.55) [57.80–89.90]	65.32 (4.76) [57.80–85.90]	68.18 (6.40) [57.80–89.90]***
Age (HUNT4)	76.82 (5.43) [70–101]	75.90 (4.68) [70–96.30]	78.59 (6.27) [70–101]***
Age (AiT)	80.23 (4.81) [73.70–100]		-
MoCA (HUNT4)	23.70 (3.39) [1–30]	24.28 (3.09) [4–30]	22.61 (3.66) [1–30]***
MoCA (AiT)	22.97 (4.06) [1–30]	22.97 (4.06) [1–30]	-
ANU-ADRI	5.52 (9.59) [–13–48]	3.63 (8.30) [–13–44]	9.09 (10.75) [–11–48]***
CAIDE	8.22 (1.73) [4.72–14.17]	8.08 (1.73) [4.72–14.17]	8.50 (1.69) [4.72–14.17]***
CogDrisk	3.33 (6.22) [−8.60–27.75]	2.12 (5.60) [−8.60–24.75]	5.59 (6.68) [−8.50–27.75]***
LIBRA	3.17 (4.43) [−6.19–23.10]	2.28 (3.94) [−6.19–20.80]	4.84 (4.81) [−6.11–23.10]***
LIBRA2	29.00 (12.45) [0–88.41]	27.19 (12.02) [0–78.90]	32.42 (12.52) [0–88.41]***
UKBDRS	11.58 (1.14) [9.53–17.09]	11.37 (0.98) [9.53–15.72]	12.00 (1.29) [9.58–17.09]***
UKBDRS-APOE	11.81 (1.22) [9.53–17.09]	11.59 (1.08) [9.53–15.72]	12.22 (1.35) [9.58–17.09]***
Lancet	13.70 (10.16) [0–63]	11.98 (8.91) [0–59]	16.94 (11.48) [0–63]***

Notes: N is the same for all risk scores; Significant differences in categorical variables assessed using Chi^2^-Tests, mean differences by groups assessed using *t*-tests. * *p* ≤ 0.05; ** *p* ≤ 0.01, *** *p* ≤ 0.001.

After coding all the risk scores, 1 939 participants had missing data in up to 5 risk score variables that led to incomplete risk scores. To retain these participants, complete risk scores were inferred by prorating observed contributions to the full theoretical scale of each risk score using the following formula: $\frac{\sum Observed\;Item\;Points}{\sum Maximum\;Possible\;Risk\;Points\;for\;Avaliable\;Items}\;\times \;Maximum\;Risk\;Score.$ The UKBDRS uses linear weights [25] for age and education, thus the theoretical maximum for this risk score was set to 100 years of age and 20 years of education.

### 2.4 Statistical analysis

Stata 18 (StataCorp) was used for all analyses. Descriptive statistics summarized HUNT3 lifestyle characteristics and examined missing data for the dementia risk scores. Differences between participants with complete and incomplete data were assessed using chi-squared and *t*-tests, with statistical significance set at *p* < 0.05.

Linear mixed-effects models (LMM) were used for all primary analyses investigating the association between cognition, cognitive decline, and the eight dementia risk scores: ANU-ADRI, CAIDE, CogDrisk, LIBRA, LIBRA2, UKBDRS, UKBDRS-APOE, and the Lancet-based risk score. LMMs were chosen because: 1) they allow for unbalanced data allowing for the inclusion of all HUNT4 70+ participants irrespective of their AiT participation status; 2) handle the varying follow-up intervals 2.9–5.2 years (*M* = 4.2, SD=0.3) between HUNT4 70+ and AiT; and 3) simulation studies suggest LMMs provide more accurate group effect estimates than alternative methods for two time points [36,37]. To ensure comparability across risk scores with different original scales and dispersions, all risk scores were z-score standardized (Mean=0, SD=1) prior to analysis. Each risk score was analyzed in separate models as both a continuous variable (z-score) and as tertiles (low, medium, high). Models included random intercepts and random slopes for each participant, and fixed effects for time (years), continuous risk score/risk group, and their interaction. Model fit was assessed using the Akaike information criterion (AIC) and likelihood ratio tests, confirming better fit when including random slopes. The primary outcomes were mean HUNT4 70+ MoCA score, and the time × risk score or group interaction (slope differences).

A second model with interaction effects for age at time of risk factor assessment (under/over 65 years at HUNT3) and sex was run on modified versions of the risk scores that excluded demographic variables (age, sex, education). This was done both to avoid collinearity issues, and also, because some risk scores (LIBRA, LIBRA2, and the Lancet-based risk score) do not include native weights for demographic variables [20].

In addition to mixed-effects models of continuous cognitive decline, we examined each risk score as a predictor of binary cognitive decline status. Logistic regression models estimated associations between standardized (z-score) risk score values and a decline of ≥3 (≈1 SD [38])MoCA points (yes/no)). A threshold of 1 SD was chosen because it likely represents real and clinically meaningful change as opposed to random variation [38]. Discriminative ability was assessed using the area under the receiver operating characteristic curve (AUC), and significant differences were assessed using DeLong’s Test. Sensitivity analyses tested alternative thresholds for decline (≥2, ≥4, ≥6, and ≥9 points) and excluded participants with low HUNT4 70+ MoCA scores (<18, <20, <22, or <24) to reduce potential floor effects.

To account for attrition between HUNT4 70+ and AiT, we applied inverse probability weighting (IPW) based on HUNT3 health characteristics. We first constructed a logistic regression model predicting participation at follow-up using the following HUNT4 70+ covariates: age, education, heart disease, depression, diabetes, hypertension, and stroke. These variables were selected as they are established predictors of dementia and mortality in older populations [13] and significantly predicted attrition in our data. The predicted probabilities of follow-up participation were extracted and used to generate IPWs as the inverse of the predicted probability for each participant. To reduce the influence of extreme weights, we truncated the upper 1 % of the weight distribution at the 99th percentile, affecting 172 observations. This approach preserves most weight variation while improving model stability and limiting bias from highly influential cases. These weights were then applied in all longitudinal mixed-effects models examining change in cognitive performance.

Model sensitivity was tested across multiple dimensions, including with and without IPW, and complete cases only.

## 3 Results

### 3.1 Descriptive statistics

A total of 7 221 HUNT4 70+ (54.1 % female) participants were included in the present study sample. The interval between risk score assessment during HUNT3 (2007–8) and HUNT4 70+ (2017–19) averaged 10.6 (SD=0.8) years, and between HUNT4 70+ and AiT (2021–23) averaged 4.2 (SD=0.3, 2.9–5.2) years. Out of 7 221 HUNT4 70+ participants, 4716 (65.3 %) also completed the AiT assessment (Table 1). At HUNT3, mean age was 66.3 (SD=5.6) years, with 49.6 % under and 50.4 % over 65 years, increasing to 76.8 (SD=5.4) years at HUNT4 70+ and 80.2 (SD=4.8) years at AiT. Non-returners were significantly older (68.2 years (SD=6.4)) than returners (65.3 years (SD=4.8)) at HUNT3; and at HUNT4 70+ (78.6 (SD=6.3) vs. 75.9 (SD=4.7), *p* < 0.001), and more likely to be ≥65 at HUNT3 (61.3 % vs. 44.5 %). Mean education was 12.7 (SD=2.3) years, with 64.9 % completing secondary and 35.0 % tertiary education. Non-returners were less likely to have tertiary education than those who returned (26.8 % vs. 39.4 %). Non-returners had overall lower cognitive scores than those who returned (22.6 (SD=3.7) vs. 24.3 (SD=3.1), *p* < 0.001). Average MoCA score was 22.97 (SD=4.1) at AiT (Table 1).

### 3.2 Risk scores predicting cognitive decline (Continous)

All eight risk scores were significantly associated with MoCA scores at HUNT4 70+ and yearly change in MoCA scores from HUNT4 70+ to AiT (Table 2; Supplementary Table 3 for full model). Higher risk scores were consistently associated with lower MoCA performance at HUNT4 70+ (ranging from β=−1.61, 95% CI:−1.72,−1.51 for UKBDRS to β=−0.74, 95% CI:−0.82,−0.67 for CAIDE) per 1SD change in risk score (all *p* < 0.001, Table 2; Supplementary Table 3). Higher risk scores were also associated with steeper yearly decline in MoCA (all *p* < 0.001). The strongest association with yearly rate of decline was seen for Lancet (β=−0.23, 95% CI:−0.27,−0.18) and UKBDRS(APOE) (both β=−0.22, 95% CI:−0.26,−0.19), followed by ANU-ADRI (β=−0.17, 95% CI:−0.21,−0.13, LIBRA (β=−0.16, 95% CI:−0.19,−0.12) and CogDrisk(β=−0.15, 95% CI:−0.19,−0.12). LIBRA2 (β=−0.07, 95% CI:−0.09,−0.04) and CAIDE (β=−0.11, 95% CI:−0.16,−0.06) showed the weakest association.

**Table 2:** Mixed effects models of continous (z-score) risk scores with, and without demographics - with ipw continuous risk scores (z-score) with demographics.

Term	ANU-ADRI β [95% CI]	CAIDE β [95% CI]	CogDrisk β [95% CI]	LIBRA β [95% CI]	LIBRA2 β [95% CI]	UKBDRS β [95% CI]	UKBDRS-APOE β [95% CI]	Lancet β [95% CI]
**MoCA Score**	23.11*** [23.02, 23.21]	23.68*** [23.60, 23.76]	23.15*** [23.06, 23.23]	23.11*** [23.02, 23.20]	23.47*** [23.38, 23.55]	23.22*** [23.13, 23.31]	23.24*** [23.15, 23.33]	23.19*** [23.10,23.29]
**Risk Score (z)**	−1.57*** [−1.67, −1.46]	−0.74*** [−0.82, −0.67]	−1.53*** [−1.63, −1.44]	−1.58*** [−1.68, −1.48]	−0.97*** [−1.06, −0.89]	−1.61*** [−1.72, −1.51]	−1.51*** [−1.62, −1.41]	−1.50*** [−1.62, −1.39]
**Time × Score (z)**	−0.17*** [−0.21, −0.13	−0.04*** [−0.07, −0.02]	−0.15*** [−0.19, −0.12]	−0.16*** [−0.19, −0.12]	−0.07*** [−0.09, −0.04]	−0.22*** [−0.26, −0.18]	−0.22*** [−0.26, −0.19]	−0.23*** [−0.27, −0.18]

Continuous Risk Scores (z-score) Without Demographics								
Term	ANU-ADRI β [95% CI]	CAIDE β [95% CI]	CogDrisk β [95% CI]	LIBRA β [95% CI]	LIBRA2 β [95% CI]	UKBDRS β [95% CI]	UKBDRS-APOE β [95% CI]	Lancet β [95% CI]
**MoCA Score**	23.73*** [23.65, 23.81]	23.80*** [23.73, 23.88]	23.70*** [23.62, 23.78]	23.66*** [23.58, 23.74]	23.67*** [23.59, 23.75]	23.82*** [23.74, 23.90]	23.82*** [23.75, 23.90]	23.72*** [23.64, 23.80]
**Risk Score (z)**	−0.47*** [−0.55, −0.38]	−0.33*** [−0.41, −0.25]	−0.56*** [−0.64, −0.49]	−0.64*** [−0.72, −0.57]	−0.63*** [−0.71, −0.55]	−0.34*** [−0.43, −0.26]	−0.25*** [−0.33, −0.17]	−0.50*** [−0.58, −0.41]
**Time × Score (z)**	−0.02 [−0.05, 0.00]	−0.03*** [−0.06, −0.01]	−0.04*** [−0.06, −0.02]	−0.03*** [−0.05, −0.01]	−0.03*** [−0.06, −0.01]	−0.06*** [−0.09, −0.04]	−0.07*** [−0.09, −0.05]	−0.04*** [−0.07, −0.02]

Notes: * *p* ≤ 0.05; ** *p* ≤ 0.01, *** *p* ≤ 0.001; All baseline participants (*n* = 7 221) and follow-up participants (*n* = 4 716) are included; MoCA Score represents the mean MoCA score at HUNT4 70+ for participants with the lowest risk profile in each risk score. Risk Score (z) represents the difference in baseline MoCA per 1 SD increase in the risk score. Time × Score (z) represents the annual change in MoCA per 1 SD increase in the risk score between HUNT4 and AiT.

When excluding demographics from the risk scores, all associations with MoCA at HUNT4 70+ remained significant, albeit with attenuated effect sizes (see Table 2). Coefficients for HUNT4 70+ MoCA scores ranged from β=−0.64 (95% CI:−0.72,−0.57) for LIBRA to β=−0.25 (95% CI:−0.33,−0.17) for UKBDRS-APOE (Table 2; Supplementary Table 3). All risk scores with the exception of ANU-ADRI, remained significantly associated with yearly slope of decline, with effect sizes ranging from β=−0.07 (95% CI:−0.09,−0.05) for UKBDRS-APOE to β=−0.02 (95% CI:−0.05, 0.00) for ANU-ADRI (Table 2; Supplementary Table 3). Sensitivity analyses using different (age, sex, education, wealth) or no IPWs, and complete-case-only analysis, showed that results did not differ substantially beyond slight changes in model fit and slight attenuation of effect sizes (Supplementary Tables 4 and 5).

### 3.3 Predicting cognitive decline by risk group

**Fig. 1:**
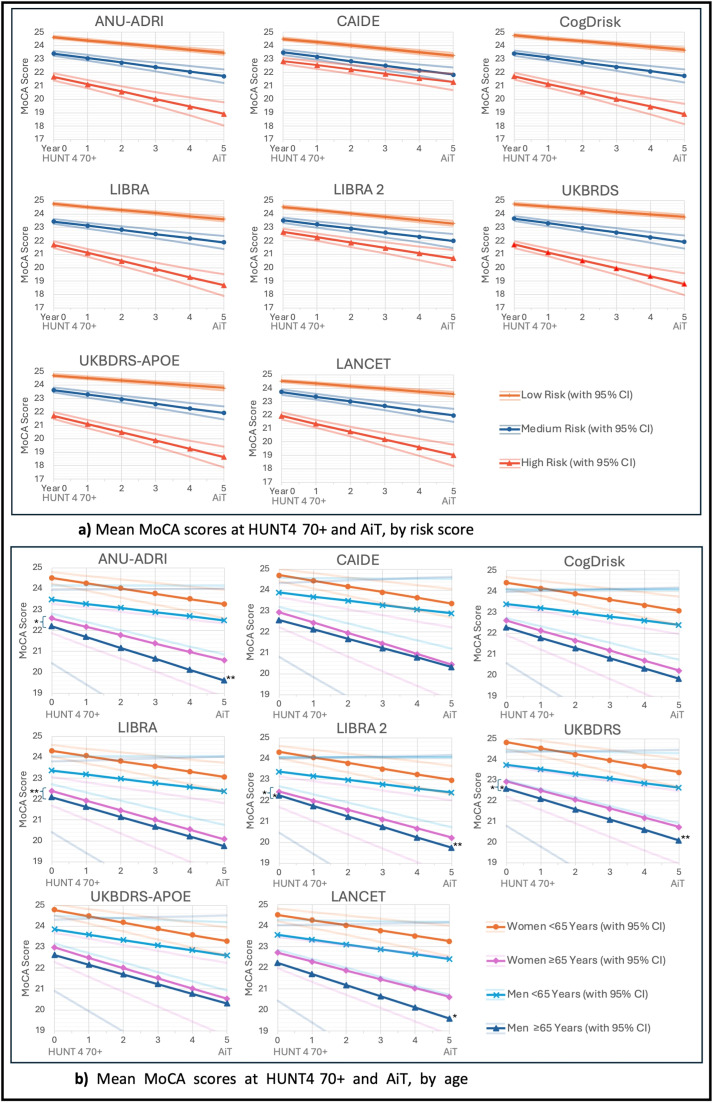
**a)** Mean MoCA scores at HUNT4 70+ and AiT, by risk score tertile group. **b)** Mean MoCA scores at HUNT4 70+ and AiT, by age group and sex.

All eight dementia risk scores measured during HUNT3 significantly predicted differences in MoCA scores at HUNT4 70+ and yearly change in global MoCA scores until AiT (Table 3; Supplementary Table 5 for full model). Higher risk categories consistently demonstrated lower MoCA performance during HUNT4 70+ and steeper rates of cognitive decline compared to low-risk participants (Fig. 1a). Medium-risk groups also differed significantly from low-risk group performance at HUNT4 70+ (ranging from β=−1.31 (95% CI:−1.47, −1.16) for CogDrisk to β=−0.83 (95% CI:−0.98, −0.67) for Lancet; all *p* < 0.001, see Table 3; Supplementary Table 5), supporting a graded association between risk burden and cognitive performance (see Fig. 1a).

**Table 3:** Mixed models of risk score terciles with, and without demographics risk score terciles without demographics.

	ANU-ADRI β [95% CI]	CAIDE β [95% CI]	CogDrisk β [95% CI]	LIBRA β [95% CI]	LIBRA2 β [95% CI]	UKBDRS β [95% CI]	UKBDRS-APOE β [95% CI]	Lancet β [95% CI]
**MoCA Score**	24.62*** [24.53, 24.72]	24.50*** [24.40, 24.61]	24.76*** [24.66, 24.85]	24.75*** [24.65, 24.84]	24.52*** [24.42, 24.63]	24.74*** [24.63, 24.84]	24.72*** [24.62, 24.82]	24.54*** [24.44, 24.65]
**High Risk (vs Low)**	−2.94*** [−3.19, −2.68]	−1.65*** [−1.86, −1.44]	−3.04*** [−3.27, −2.81]	−3.04*** [−3.27, −2.80]	−1.86*** [−2.08, −1.65]	−3.02*** [−3.25, −2.79]	−3.00*** [−3.24, −2.77]	−2.61*** [−2.85, −2.37]
**Time × High Risk**	−0.32*** [−0.43, −0.22]	−0.07* [−0.13, 0.00]	−0.35*** [−0.44, −0.26]	−0.38*** [−0.47, −0.28]	−0.15*** [−0.22, −0.08]	−0.40*** [−0.49, −0.31]	−0.43*** [−0.52, −0.34]	−0.39*** [−0.48, −0.30]

Risk Score Terciles Without Demographics								
	ANU-ADRI β [95% CI]	CAIDE β [95% CI]	CogDrisk β [95% CI]	LIBRA β [95% CI]	LIBRA2 β [95% CI]	UKBDRS β [95% CI]	UKBDRS-APOE β [95% CI]	Lancet β [95% CI]
**MoCA Score**	24.21*** [24.10, 24.31]	24.06*** [23.95, 24.16]	24.31*** [24.20, 24.43]	24.37*** [24.26, 24.48]	24.38*** [24.27, 24.49]	24.10*** [23.99, 24.21]	24.12*** [24.00, 24.25]	24.23*** [24.12, 24.34]
**High Risk (vs Low)**	−0.90*** [−1.09, −0.70]	−0.69*** [−0.88, −0.51]	−1.15*** [−1.36, −0.95]	−1.36*** [−1.56, −1.16]	−1.35*** [−1.54, −1.15]	−0.61*** [−0.78, −0.43]	−0.49*** [−0.67, −0.30]	−1.05*** [−1.25, −0.86]
**Time × High Risk**	−0.03 [−0.09, 0.03]	−0.06* [−0.12, −0.01]	−0.10** [−0.16, −0.04]	−0.05 [−0.11, 0.01]	−0.04 [−0.10, 0.01]	−0.08** [−0.13, −0.03]	−0.15*** [−0.20, −0.10]	−0.10** [−0.15, −0.04]

Notes: * *p* ≤ 0.05; ** *p* ≤ 0.01, *** *p* ≤ 0.001; All baseline participants (*n* = 7 221) and follow-up participants (*n* = 4 716) are included; MoCA Score represents the mean MoCA score at HUNT4 70+ for participants in the low-risk tertile per risk score. High Risk (vs Low) represents the difference in MoCA at baseline between high- and low-risk tertiles. Time × High Risk represents the annual change in MoCA for participants in the high-risk tertile compared with the low-risk tertile.

CogDrisk and LIBRA showed the largest differences in HUNT4 70+ MoCA scores between high- and low-risk groups (β=−3.04, 95% CI:−3.27,−2.81, and β=−3.04, 95% CI:−3.27,−2.80, respectively), followed closely by UKBDRS (β=−3.02, 95% CI:−3.25,−2.79), UKBDRS-APOE (β=−3.00, 95% CI:−3.24,−2.77), ANU-ADRI (β=−2.94, 95% CI:−3.19,−2.68), and Lancet (β=−2.61, 95% CI:−2.85,−2.37). LIBRA 2(β=−1.86, 95% CI:−2.08 to −1.65) and CAIDE (β=−1.65, 95% CI:−1.86 to −1.44) yielded smaller high-risk group differences in HUNT4 70+ MoCA scores (Table 3; Fig. 1a).

All high-risk group differences and risk group × time interactions were statistically significant (*p* < 0.001). UKBDRS-APOE (β=−0.43, 95% CI:−0.52 to −0.34), UKBDRS (β=−0.40, 95% CI:−0.49 to −0.31), the Lancet-based risk score (β=−0.39, 95% CI:−0.48 to −0.30), LIBRA (β=−0.38, 95% CI:−0.47 to −0.28), CogDrisk (β=−0.35, 95% CI:−0.44 to −0.26), and ANU-ADRI (β=−0.32, 95% CI:−0.43 to −0.22) were associated with the steepest yearly rates of decline in the high-risk group, followed by LIBRA2(β=−0.15, 95% CI:−0.22 to −0.08), with CAIDE showing the smallest slope difference (β=−0.07, 95% CI:−0.13 to 0.00) (Table 3, Fig. 1a). Medium risk x time interactions were also significant for all risk scores but with less steep slopes compared to high risk, ranging from β=−0.16 (95% CI:−0.20,−0.11) for UKBDRS(APOE) to β=−0.06 (95% CI:−0.11, 0.01) for LIBRA2 (Supplementary Table 5; Fig. 1a).

Excluding demographics from the risk scores attenuated effect sizes. High-low risk group separation at HUNT4 70+ remained significant for all scores (Table 3), ranging from β=−1.36 and-1.35 (95% CI:−1.54,−1.16) for LIBRA and LIBRA2 to β=−0.49 (95% CI:−0.67,−0.30) for UKBDRS-APOE. Medium-low risk group separation remained significant for all risk scores except CAIDE (see Supplementary Table 5). Significant associations with yearly slopes of decline at medium risk were observed only for UKBDRS (β=−0.09, 95% CI:−0.15,−0.03), CAIDE (β=−0.08, 95% CI:−0.13,−0.02), CogDrisk (β=−0.07, 95% CI:−0.12,−0.02), and UKBDRS-APOE (β=−0.07, 95% CI:−0.12,−0.02) (Supplementary Table 5). At high risk, significant associations with yearly slope of decline were found only for UKBDRS-APOE (β=−0.15, 95% CI:−0.20,−0.10), Lancet (β=−0.10, 95% CI:−0.15,−0.04), CogDrisk (β=−0.10, 95% CI:−0.16,−0.04), UKBDRS (β=−0.08, 95% CI:−0.13,−0.03), and CAIDE (β=−0.06, 95% CI:−0.12). Sensitivity analyses using different or no IPWs, and complete case only analysis, showed that results did not differ substantially beyond slight changes in model fit and slight attenuation of effect sizes (Supplementary Tables 6 and 7).

### 3.4 Risk scores predicting cognitive decline by age and sex

Significant age-related differences in the association between continuous dementia risk scores and cognition at HUNT4 70+ were observed for several risk scores (Supplementary Table 8; Fig. 1b). For older adults (≥65 years at HUNT3), the negative association with HUNT4 70+ cognitive score was most pronounced for LIBRA (β=−0.31, 95% CI:−0.52,−0.10, *p* < 0.01) and UKBDRS (β=−0.29, 95% CI:−0.53,−0.06, *p* < 0.05), followed by LIBRA2 (β=−0.25, 95% CI:−0.47,−0.03, *p* < 0.05) and ANU-ADRI (β=−0.25, 95% CI:−0.46,−0.03, *p* < 0.05). For UKBDRS and LIBRA2 men over 65 years sowed significantly higher MoCA scores at HUNT4 70+ (β=0.35, 95% CI:0.01,0.69, and β=0.32, 95% CI:−0.02,0.62, respectively; Supplementary Table 8). Age- and sex-related differences in cognitive decline were observed only among older men, who exhibited steeper decline for UKBDRS (β=−0.15, 95 %CI:−0.25,−0.05, *p* < 0.01), ANU-ADRI (β=−0.15, 95% CI:−0.25,−0.05, *p* < 0.01), LIBRA2 (β=−0.13, 95% CI:−0.23,−0.03, *p* < 0.01, and Lancet (β=−0.13, 95% CI:−0.22,−0.03, *p* < 0.05) risk scores. No other significant age- or sex-related differences in cognition at HUNT4 70+ or cognitive decline were observed for any risk score. However, visual inspection of predicted trajectories suggested some group separation beyond what was statistically supported (Fig. 1b). Sensitivity analyses using different or no IPWs, and complete case only analysis, showed that results did not differ substantially beyond slight changes in model fit and slight attenuation of effect sizes (Supplementary Tables 9 and 10).

### 3.5 Predicting cognitive decline as a binary outcome

All eight dementia risk scores were significantly associated with cognitive decline, defined as *a* ≥ 3-point (1 SD) reduction in cognitive score between HUNT4 70+ and AiT (Table 4). However, overall discriminative ability was modest. UKBDRS (β=0.69, 95% CI:0.59,0.80; AUC=0.61), UKBDRS-APOE (β=0.68, 95% CI:0.58,0.78; AUC=0.61), CogDrisk (β=0.58, 95% CI:0.49,0.67; AUC=0.60), and Lancet (β=0.66, 95% CI:0.54,0.77; AUC=0.60) demonstrated the strongest performance, followed by ANU-ADRI (β=0.60, 95% CI:0.50,0.70; AUC=0.59), LIBRA (β=0.57, 95% CI:0.47–0.67; AUC=0.59), LIBRA2 (β=0.29, 95% CI:0.21–0.36; AUC=0.56), and CAIDE (β=0.19, 95% CI:0.13–0.25; AUC=0.55). Notably, none of the risk scores outperformed a demographics-only model (age, education; AUC=0.61), with CAIDE, LIBRA, LIBRA2, and ANU-ADRI performing significantly worse (DeLong *p* < 0.05).

**Table 4:** Logistic regression of continuous risk score predicting binary cognitive change With Demographics (MOCA1 (Change *n* = 1 488; No Change 1SD/3pts=3 228).

Risk Score	Coefficient	95% CI	AIC	ROC AUC	ROC 95% CI
**Age**	0.08***	0.07–0.10	8821.04	0.61	0.60–0.63
**Education**	−0.32***	−0.45–0.19			
**ANU-ADRI**	0.60***	0.50–0.70	8905.69	0.59*	0.58–0.61
**CAIDE**	0.19***	0.13–0.25	9123.22	0.55***	0.54–0.57
**CogDrisk**	0.58***	0.49–0.67	8893.41	0.60	0.58–0.62
**LIBRA**	0.57***	0.47–0.67	8910.88	0.59*	0.57–0.61
**LIBRA2**	0.29***	0.21–0.36	9080.24	0.56***	0.54–0.58
**UKBDRS**	0.69***	0.59–0.80	8831.72	0.61	0.59–0.63
**UKBDRS-APOE**	0.68***	0.58–0.78	8832.47	0.61	0.60–0.63
**Lancet**	0.66***	0.54–0.77	8884.40	0.60	0.58–0.61

No Demographics Included					
Risk Score	Coefficient	95% CI	AIC	ROC AUC	ROC 95% CI
**ANU-ADRI**	0.11***	0.04–0.17	9163.98	0.52	0.51–0.54
**CAIDE**	0.09***	0.03–0.16	9165.61	0.53	0.51–0.54
**CogDrisk**	0.18***	0.11–0.24	9133.83	0.54	0.52–0.56
**LIBRA**	0.14***	0.08–0.21	9147.72	0.53	0.51–0.55
**LIBRA2**	0.15***	0.08–0.22	9147.57	0.53	0.51–0.55
**UKBDRS**	0.16***	0.09–0.23	9146.07	0.53	0.52–0.55
**UKBDRS-APOE**	0.15***	0.08–0.22	9146.70	0.54	0.52–0.56
**Lancet**	0.16***	0.09–0.23	9146.82	0.53	0.52–0.55

Notes: Notes: (below (*n* = 3 228) /above (*n* = 1 488) 3 points (i.e. 1 SD) MoCA change over 4 years), models using IPW on all participants with both baseline and follow-up MoCA score (*n* = 4 595). * *p* ≤ 0.05; ** *p* ≤ 0.01, *** *p* ≤ 0.001.

Excluding demographic variables resulted in a further reduction in predictive performance across all risk scores (AUC range: 0.52–0.54). CogDrisk (β=0.18, 95% CI:0.11–0.24; AUC=0.54) remained the strongest, followed by UKBDRS-APOE (β=0.15, 95% CI:0.08–0.22; AUC=0.54), UKBDRS (β=0.16, 95% CI:0.09–0.23; AUC=0.53), Lancet (β=0.16, 95% CI:0.09–0.23; AUC=0.53), LIBRA (β=0.14, 95% CI:0.08–0.21; AUC=0.53), LIBRA2 (β=0.15, 95% CI:0.08–0.22; AUC=0.53), CAIDE (β=0.09, 95% CI:0.03–0.16; AUC=0.53), and ANU-ADRI (β=0.11, 95% CI:0.04–0.17; AUC=0.52). In all cases, risk scores without demographics performed significantly worse than the demographics-only model (all DeLong *p* < 0.001, Table 4).

Sensitivity analyses excluding participants with low HUNT4 70+ cognitive scores (<18, <20, <22, or <24) to account for potential floor effects did not improve discrimination across risk scores. Neither did changing the definition for cognitive change to a MoCA change of 2 or 4 points. Additional analyses using *a* ≥ 2 SD and ≥3 SD (i.e., ≥6 (Supplementary Table 11) and ≥9 point (Supplementary Table 12)) cognitive change as the endpoint showed similar rank ordering of risk scores, with AUCs increasing by approximately 0.02–0.1 points.

## 4 Discussion

To our knowledge, this study is the first to investigate, within the same sample, associations between cognition, cognitive decline and eight widely used dementia risk scores; ANU-ADRI, CAIDE, CogDrisk, LIBRA, LIBRA2, UKBDRS, UKBDRS-APOE, and a Lancet-based score. Across all risk scores, higher risk was consistently associated with lower MoCA scores at HUNT4 70+ and faster subsequent decline. The largest separations in cognitive function at HUNT4 70+ were observed for CogDrisk and LIBRA, both when scores were modeled continuously and in group-based comparisons, followed by LIBRA2, ANU-ADRI, UKBDRS, and the Lancet-based score. CAIDE and UKBDRS-APOE showed smaller differences at HUNT4. With respect to decline, UKBDRS-APOE and UKBDRS showed the steepest yearly high-risk group declines, followed by the Lancet-based risk score, LIBRA, CogDrisk, and ANU-ADRI; LIBRA2 and CAIDE showed smaller slope separations. When modeled continuously, CogDrisk and LIBRA demonstrated the strongest associations with both cognitive function and decline, followed by LIBRA2 and ANU-ADRI, with UKBDRS, UKBDRS-APOE, Lancet, and CAIDE showing smaller effect sizes. Removing age, sex and education significantly decreased effects across risk scores and worsened fit. All risk scores, with the exception of CAIDE, exhibited age-related differences in the association between risk score and cognition at HUNT4. Significant age- and sex-related differences in cognitive decline were observed only among older men, who exhibited steeper decline for UKBDRS, ANU-ADRI, LIBRA2, and Lancet-based risk scores. No other significant age- or sex-related differences in cognition or cognitive decline were observed for any risk score.

A comparison of the risk scores in their ability to classify binary ≥3-point (1 SD) cognitive decline assessed by MoCA showed only modest discriminatory ability, with UKBDRS and UKBDRS-APOE performing best, closely followed by the Lancet-based risk score and CogDrisk; ANU-ADRI and LIBRA were slightly lower, while LIBRA2 and CAIDE showed the weakest discrimination. However, no risk score outperformed a model including age and education alone. Furthermore, removing age and education from the risk scores reduced their discriminatory ability to only marginally better than chance (AUC=0.52–0.54), highlighting the key role of these demographic characteristics in risk prediction. These findings were closely mirrored in a recent study comparing ANU-ADRI, CogDrisk, CAIDE, and LIBRA in their ability to predict mild cognitive impairment [39]. Here too, risk scores without demographic variables achieved AUCs barely better than chance [39]. Further, these results mirror previous findings from the same population within the HUNT cohort, which showed that no risk score clearly outperformed a simple demographics model (age + education) in dementia risk prediction, though with better discriminatory ability overall (AUCs 0.54–0.78) [23]. AUC scores improved by about 0.03 points when decline was defined as *a* ≥ 6 MoCA point change (≈2 SD) between HUNT4 70+ and AiT, and rose above 0.7 when defining decline as ≥9 point change (≈3 SD). However, a 6-point MoCA decline over a 4-year interval is uncommon in community cohorts (about 10 % in this cohort) and more consistent with severe pathology, so these thresholds are less suitable for routine monitoring [35,38].

Given that the eight risk scores were initially developed to assess dementia risk at the individual or population level, it is not surprising that effect sizes for measuring cognitive decline are lower. Whereas dementia endpoints are more stable and reliant on multiple points of evidence [34], MoCA’s bounded scale and potential practice- and floor/ceiling effects, may compress variance, lower separability and increase outcome misclassification. Even more than a dementia diagnosis, which relies on other clinical markers, cognitive decline may be masked by higher cognitive reserve. Cognitive reserve is often associated with higher education [40], making education even more important in these risk scores when measuring cognitive decline instead of dementia. This is supported by the fact that the coefficient for education is four times higher than for age in this study, while it was half that of age when assessing dementia risk in the same cohort [23]. CAIDE and LIBRA2 being among the weakest and UKBDRS being among the strongest performing risk scores but achieving effect sizes not significantly different from any other risk score when removing age and education, further highlight the importance of age and education, and the attention that should be paid to both the inclusion of these risk factors and *how* they are included. CAIDE is well known for achieving poor discriminatory ability in older populations as it assigns the same weight to everyone over 53 years, thus losing age-dependent nuance [22,23,41]. UKBDRS, on the other hand, attributed continuous weighting per year of age and education, thus achieving higher effect sizes and better separation of risk groups. By contrast, the Lancet-based risk score, including age and education, produced more modest contrasts, but the best overall model fit, underscoring its strength as an efficient, population-oriented composite rather than a risk score designed to maximize individual-level separation. This reflects that the Lancet-based score was operationalized from a population-level framework and has not yet undergone external validation as an individual-level risk index.

The different measures of cognition and decline, together with comparisons of AUC values, highlighted both strengths and weaknesses of the various risk scores. This underlines that score selection should be made carefully, as different users face different optimization problems. For risk prediction, IPW or other tasks that require continuous risk scores, CogDrisk or LIBRA might be the strongest contenders. For trial selection or individual risk communication, tools that yield larger high- vs. low-risk contrasts and slightly higher discrimination, such as CogDrisk, ANU-ADRI, or UKBDRS, or Lancet, may be more suitable. At the same time, individuals seeking to understand and reduce their own modifiable risks may benefit most from risk scores with broad coverage of lifestyle and health factors, such as CogDrisk, ANU-ADRI, or LIBRA(2). Researchers and policymakers may favour UKBDRS, given its strong adherence to register-based data (missingness was only 2.6 % for this risk score, compared with an average of 27 % for other scores in this study). Ultimately, our findings reinforce that these instruments are most valuable as structured profiles of modifiable risk, supporting tailored advice and behavior/policy change, rather than statistical tools for risk modeling of cognitive decline. It is also important to note that these risk scores were developed for dementia risk prediction, and while cognitive decline is an important precursor for dementia, it is not surprising that these risk scores display lower associations with cognitive decline [39].

### 4.1 Strengths and limitations

To our knowledge, this is the first within-cohort, harmonized comparison spanning seven commonly used dementia risk scores and a purpose-made Lancet-based risk score. It is also the first study comparing all eight dementia risk scores in their ability to predict cognition 10.6 years after lifestyle assessment and cognitive decline 4.2 years later. The 15-year span of this study also makes this one of the studies with the longest follow-up times in this field [11]. Additionally, this study comprises data from 7 221 participants at the first cognitive assessment at HUNT4 70+ and 4716 at follow-up at AiT four years later, representing nearly 40 % and nearly one quarter of the regional population aged 70+ [34], respectively. Given this large sample size and the continuous nature of our outcome variable (MoCA score), this study had sufficient power to compare the eight risk scores across multiple conditions, even despite small effect sizes [42]. This study benefits from the many overall strengths of the HUNT Study (discussed in detail here [23,32]), including, but not limited to, the ability to cross-link participants with other national registries, allowing for near complete risk score generation, even in the case of UKBDRS, which uses financial and domestic risk factors.

Despite this, the study faces some limitations. As commonly reported in aging cohorts [32], this study faces substantial attrition: only 65.3 % of the HUNT4 70+ sample returned for follow-up. Exploratory analyses indicated that non-returners were in poorer health, had lower baseline cognitive scores, and were disproportionately represented in the highest risk groups (this pattern is described in more detail in previous studies [23,33]). This selective attrition likely attenuated observed group differences, particularly among high-risk participants. We applied IPW and conducted multiple sensitivity analyses to mitigate this bias, but residual effects cannot be fully excluded.

Additionally, our study includes cognitive measures at only two time points, which has been argued to be insufficient for adequate change modeling [36]. To counteract this, we used mixed-effects models to estimate group-level changes in cognitive performance. Simulation evidence suggests that while two-point models cannot reliably capture individual differences in change, they can recover average fixed effects with reasonable accuracy [37]. Despite this, the limited number of repeated measures may partly explain why our study found little statistical support for interaction terms on slopes for age and sex, even though prior work reported attenuating effects of sex and age group on risk score performance [23].

To expand on this limitation, the use of global MoCA change may mask true effects due to floor and ceiling effects, regression to the mean, relatively small possible variations in scores and domain-specific sensitivity (e.g., executive function, attention, memory); as well as possible practice effects which may mask true change. Future studies should assess other, more sensitive and more domain-specific cognitive measures. Some risk score components required proxy variables (e.g. HUNT-specific proxies for cognitive activity (see Supplementary Table 2)), or omitted items (e.g. pesticide exposure, chronic kidney disease, vision loss), introducing possibility for misclassification. Additionally, the Lancet-based score has not been externally validated. These limitations combined suggest that true effects may have been underestimated, and should be interpreted cautiously before consideration for clinical application.

### 4.2 Conclusion

In this study, all eight dementia risk scores captured meaningful gradients in cognitive level and decline, 10.6 and 14.8 years after the initial risk assessment. Nevertheless, none achieved strong individual-level discrimination for short-term decline, and none outperformed a simple model including age and education only. The optimal choice of risk score depends on purpose, with UKBDRS, ANU-ADRI and LIBRA showing the strongest association with cognition at HUNT4 70+, and UKBDRS and the Lancet-based risk score showing the strongest association with 4-year decline between HUNT4 70+ and AiT. However differences are marginal and effect sizes are small. Overall, our findings suggest that the risk scores studied here are well suited to describe an individual’s risk profile to inform prevention or general risk classification but have limited utility as statistical tools for risk prediction or quantitative assessment.

## Funding

This project is funded by the Norwegian Research Council (303419).

The genotyping was financed by the National Institute of health (NIH), University of Michigan, The Norwegian Research Council, and Central Norway Regional Health Authority and the Faculty of Medicine and Health Sciences, Norwegian University of Science and Technology (NTNU). The genotype quality control and imputation has been conducted by the K. G. Jebsen center for genetic epidemiology, Department of public health and nursing, Faculty of medicine and health sciences, Norwegian University of Science and Technology (NTNU). GCF is funded by the Faculty of Medicine and Health Sciences at NTNU and Central Norway Regional Health Authority.

## Data sharing

The data used in this study is owned by the HUNT research center, Statistics Norway, the Norwegian Patient Registry and the Norwegian Cause of Death Registry. Access requires approval from the Regional Ethics Committees and the data owners. The authors are not permitted to share the data with third parties but can be contacted with questions.

## Declaration of generative AI and AI-Assisted technologies in the writing process

During the preparation of this work, the author(s) used ChatGPT (OpenAI) and Microsoft Copilot to provide critical feedback on clarity and language. No AI-generated content was inserted directly, and the author(s) take full responsibility for the content of the publication.

## CRediT authorship contribution statement

**Josephine Stubs:** Writing – review & editing, Writing – original draft, Validation, Project administration, Methodology, Formal analysis, Data curation, Conceptualization. **Geir Selbæk:** Writing – review & editing, Supervision, Resources, Project administration, Methodology, Funding acquisition. **Bjørn Heine Strand:** Writing – review & editing, Methodology, Funding acquisition. **Gill Livingston:** Writing – review & editing, Supervision, Methodology, Funding acquisition, Conceptualization. **Kaarin J. Anstey:** Writing – review & editing, Methodology, Funding acquisition, Conceptualization. **Kay Deckers:** Writing – review & editing, Methodology, Conceptualization. **Mika Kivimäki:** Writing – review & editing, Methodology, Funding acquisition, Conceptualization. **Steinar Krokstad:** Writing – review & editing, Funding acquisition, Data curation, Conceptualization. **Fiona E. Mathews:** Writing – review & editing, Methodology, Conceptualization. **Ellen Melbye Langballe:** Writing – review & editing, Supervision, Resources, Methodology, Formal analysis.

## Declaration of competing interest

The authors declare that they have no known competing financial interests or personal relationships that could have appeared to influence the work reported in this paper.

## Appendix Supplementary materials

Image, application 1
