# Supplementary material for: Predicting cognitive decline: Comparative analysis of ANU-ADRI, CAIDE, CogDrisk, LIBRA, LIBRA2, UKBDRS and Lancet based dementia risk scores in the HUNT study
Source: J Prev Alzheimers Dis. 2026 Feb 28;13(4):100524. doi: 10.1016/j.tjpad.2026.100524 (PMC12966730; doi:10.1016/j.tjpad.2026.100524)
Supplement: Supplementary file 1 [file mmc1.pdf]

Supplementary

INTRODUCTION

Supplemental Table 1) Risk score overview and weighting

	ANU-ADRI	CAIDE	CogDrisk	LIBRA	LIBRA2	LANCET	UKBDRS
**Development methodology**	Evidence-Based Medicine approach. Initial list of 38 factors identified through systematic reviews and literature search; refined based on strength of evidence.	Derived from the Finnish CAIDE cohort by linking midlife cardiovascular and lifestyle data (1972-1987) with dementia diagnoses at 20-year follow-up (1998). Logistic regression identified midlife risk factors predictive of late-life dementia.	Evidence-Based Medicine approach. Comprehensive systematic review of meta-analyses of observational studies to identify risk/protective factors for dementia and subtypes.	Mixed-method design combining a systematic literature review with a Delphi expert consensus study. PubMed search identified candidate factors; Delphi panel of 8 experts ranked modifiable dementia risk/protective factors.	Update of LIBRA using an umbrella review and a second Delphi expert consensus. Expanded factor list to reflect new evidence on modifiable risks.	Systematic reviews, meta-analyses, Mendelian randomization, triangulation across study types	Developed in UK Biobank with Whitehall II validation. 28 candidate variables were compiled from meta-analyses and Lancet Commission factors, restricted to variables available in UK Biobank and measurable in primary care. Final variables were selected using LASSO Cox regression
**Inclusion criteria**	Study inclusion criteria >100 participants, >12 months follow-up, accepted AD diagnostic criteria). Only Factors measurable via self-report	Candidate risk factors were previously implicated in dementia or vascular disease. Variables were tested in logistic regression models; those showing significant associations with incident dementia were retained.	Factors required systematic review-level evidence with risk ratios for dementia, feasibility of assessment across settings (preferably via self-report), and for pharmacological exposures, supportive evidence from both cohort and trial data.	Prospective observational studies with ≥200 participants, age ≥45, ≥1 year follow-up. Only modifiable factors relevant to lifestyle intervention.	Same as LIBRA	Risk factors with consistent, high-quality evidence; dose-response; presence ≥10 years before dementia onset, modifiable by public heath intervention	Predictors required (1) consistent association with dementia in prior studies, (2) availability in UK Biobank, and (3) feasibility of collection in primary care and from registries. Highly correlated variables (r≥0.8) were excluded to avoid collinearity.
**Weighting approach**	Pooled odds ratios (ORs) from meta-analyses or cohort studies converted into standardized beta-weights	Logistic regression coefficients for each factor standardized and rounded to integers; lowest β set to ~1.	Risk ratios (RRs) extracted from umbrella review or pooled from cohort studies. Converted into points that sum to an overall risk score	Weights initially based on risk estimates from published meta-analyses. In Delphi round 2, experts distributed 100 points across factors to indicate importance	All original and new factors re-weighted based on recent meta-analytic risk estimates, with updated standardized scoring.	No individual-level risk score was developed, pooled hazard ratios were reported and Population Attributable Fractions (PAFs) were calculated for each risk factor using global prevalence and relative risk estimates. In this study individual hazard ratios are used for weighting (see below).	Variable selection via LASSO Cox regression; Retained predictors were entered into a competing risk model to derive weights, accounting for death as a competing risk.
**Primary Use**	Prediction of total dementia risk at the individual and population level. Designed for online self-assessment, intervention and epidemiological screening.	Prediction of total dementia risk from midlife profiles. Used in epidemiology, stratification, and early prevention.	Prediction of total dementia risk (all dementia and AD subtypes). Used for clinical- and self-assessment via online questionnaire.	Profiling of modifiable dementia risk factors for prevention. Designed to inform individual risk-reduction strategies and interventions.	Same as LIBRA.	Framework for modifiable dementia risk at the population level. Provides population attributable fractions for prevention planning and policy guidance.	Prediction of total dementia risk optimized for parsimonious use in registry- and cohort-based research, scalable to primary care.
**Variables**							
**High Cholesterol**	No = 0; Yes = 3	No = 0; Yes = 1	No = 0; Yes = 3	No = 0; Yes = 1.6	No = 0; Yes = 3.5	No = 0; Yes = 1.3	No = 0; Yes = 0.104
**BMI**	18.5-24.99 = 0; 25-29.99 = 2; ≥30 =5	<30 = 0 ; ≥30 = 2	<18.5 = 2; 18.5-24.99 = 0; 25-29.99 = 2; ≥30 =5"	<30 = 0 ; ≥30 = 1.6	<30 = 0 ; ≥30 = 7	<30 = 0 ; ≥30 = 1.3	-
**Depression**	No = 0; Yes = 2	-	No = 0; Yes = 3	No = 0; Yes = 2.1	No = 0; Yes = 13	No = 0; Yes = 2.2	No = 0; Yes = 0.556
**Diabetes**	No = 0; Yes = 3	-	No = 0 (M), 0 (F); Yes = 2 (M), 3 (F)	No = 0; Yes = 1.3	No = 0; Yes = 6.8	No = 0; Yes = 1.7	No = 0; Yes = 0.536
**Hypertension**	-	No = 0; Yes = 2	No = 0; Yes = 1	No = 0; Yes = 1.6	No = 0; Yes = 3.5	No = 0; Yes = 1.2	No = 0; Yes = 0.159
**Physical Activity**	Low = 0; Medium = -2; High = -3;	Low = 1; Medium = 0; High = 0	Low = 0; Medium = -3; High = -3;	Low = 1.1; Medium = 0; High = 0	Low = 6; Medium = 0; High = 0	Low = 1.2; Medium = 0; High = 0	-
**Education**	<8y = 6; 8-11y = 3; >11y = 0	<8y = 4; 8-11y = 3; >11y = 0	<8y = 4; 8-11y = 2; >11y = 0	(<8y = 2.7; 8-11y = 1.4; >11y = 0)	(<8y = 2.7; 8-11y = 1.4; >11y = 0)	<8y = 1.6; 8-11y = 0; >11y = 0	-0.041 * Years of Education
**Smoking**	No = 0; Former Smoker = 1; Yes = 4	-	No = 0; Yes = 1	No = 0; Yes = 1.5	No = 0; Yes = 7.9	No = 0; Yes = 1.3	-
**Cognitive Activity**	Low = 0; Medium = -6; High = -7	-	Low = 0; Medium = -4; High = -5	Low = 0; High = -3.2	Low = 9.4; High = 0	-	-
**Social Activity**	Highest = 0; Medium to High = 1; Low to Medium = 4; Lowest = 6	-	Not Lonely= 0; Lonely = 2	-	Highest = 0; Lowest = 6.5	No Social Isolation= 0; Social Isolation = 1.6	Does Not Live Alone = 0; Lives Alone = 0.141
**Age**	<65y = 0 (M), 0 (F); 65-69y = 1 (M), 5 (F); 70-74 = 12y (M), 14 (F); 75-79y = 18 (M), 21 (F); 80-84y = 26 (M), 29 (F); 85-89y = 33 (M), 35 (F) >90y = 38 (M), 41 (F)	<47y = 0; 47-53y = 3; >53y = 5	<65y = 0 (M), 0 (F); 65-69y = 6 (M), 4 (F); 70-74y = 8 (M), 7 (F); 75-79y = 13 (M), 11 (F); 80-84y = 17 (M), 15 (F); 85-89y = 20 (M), 19 (F) >90y = 22 (M), 23 (F)	(<65y = 0 (M), 0 (F); 65-69y = 0.4 (M), 2.1 (F); 70-74y = 5.2 (M), 6.2 (F); 75-79y = 6.8 (M), 9.2 (F); 80-84y = 11.2 (M), 12.4 (F); 85-89y = 14.1 (M), 15.3 (F) >90y = 16.4 (M), 17.6 (F))	(<65y = 0 (M), 0 (F); 65-69y = 0.4 (M), 2.1 (F); 70-74y = 5.2 (M), 6.2 (F); 75-79y = 6.8 (M), 9.2 (F); 80-84y = 11.2 (M), 12.4 (F); 85-89y = 14.1 (M), 15.3 (F) >90y = 16.4 (M), 17.6 (F))	-	0.178* (Age -50years)
**Alcohol Use**	None = 0; Light-Moderate = -3; Excessive = 0	-	-	None = 0; Light-Moderate = -1; Excessive = 0	None = 0; Light-Moderate = -0; Excessive = 3.1	None = 0; Light-Moderate = -0; Excessive = 1.2	-

**Healthy Diet**	Fish Intake: 0-0.25 servings/wk = 0; 0.26-2 servings/wk = -3; 2.1-4 servings/wk = -4; >4.1 servings/wk = -5	-	Fish Intake: 0-0.25 servings/wk = 0; 0.26-2 servings/wk = -0.25; 2.1-4 servings/wk = -0.25; >4.1 servings/k = -0.25	Diet: Mediterranean: Yes = -1.7; No = 0	Diet: Mediterranean: Yes = 0; No = 3.8	-	-
**Sex**	Included in age		Included in age and diabetes	-	-	-	Female = 0; Male = 0.169
**CHD**	-	-	No = 0; Yes = 2	No = 0; Yes = 1	No = 0; Yes = 8.3	-	-
**TBI**	No = 0; Yes = 4	-	No = 0; Yes = 2	-	-	No = 0; Yes = 1.7	-
**APOE ε4 Carrier**	-	No = 0; Yes = 2	-	-	-	-	(No = 0; Yes = 1.129)
**CKD**	-	-	-	No = 0; Yes = 1.1	No = 0; Yes = 5.7	-	-
**Hearing**	-	-	-	-	Normal = 0; Impaired = 7.6	Normal = 0; Impaired = 1.4	-
**Environment**	No Pesticide Exposure = 0; Pesticide Exposure = 2	-	-	-	-	No Air Pollution = 0; Air Pollution = 1.1	-
**Sleep Problems**	-	-	No = 0, Yes = 2	-	No = 0; Yes = 3.3	-	-
**Stroke History**	-	-	No = 0; Yes = 2	-	-	-	No = 0; Yes = 0.655
**Material Deprivation**	-	-	-	-	-	-	No = 0; Yes = 0.228
**Parental History of Dementia**	-	-	-	-	-	-	No =: Yes = 0.431
**Vision Loss**	-	-	-	-	-	No = 0; Yes = 1.5	-

Notes: Overview of dementia risk prediction frameworks, their methods, development and included variables. Body Mass Index (BMI), Chronic Heart Disease (CHD), Traumatic Brain Injury (TBI), chronic kidney disease (CKD); Variables in Parentheses (Age and Education for LIBRA and LIBRA2; APOE Status for UKBDRS) are not included in all iterations of the risk score.

METHOD

Supplementary Table 2) Overview of variable collection and coding

Variable (Risk Score)	Data Source and Register Variable Code	Methods
**Alcohol consumption** *(ANU-ADRI, LIBRA, LIBRA2, CogDrisk, Lancet; not in CAIDE)*	HUNT3: AlcTotUnitW_NT3BLQ1	Units of weekly alcohol consumed were measured using self-report data. The HUNT survey defines units of alcohol in as equal to 1) 33 cl beer (4.5%) = 11,9 g of pure alcohol 2) 15 cl wine (12%) = 14.4 g of pure alcohol 3) 4 cl spirits (40%) = 12.8 g of pure alcohol. Units/week recoded: 0 = “low”, 1–13 = “low–moderate”, ≥ 14 = “excessive”.
**APOE ε4 status** *(CAIDE; UKBDRS)*	HUNT genome panel (Illumina HumanCoreExome): SNPs rs429358, rs7412	DNA from HUNT participants was analyzed using Illumina HumanCoreExome arrays. APOE genotyping used rs429358 and rs7412 single nucleotide polymorphisms[1]. Presence of ≥ 1 ε4 allele (ε2/ε4, ε3/ε4, ε4/ε4) was coded ε4-positive = 1; all other diplotypes ε4-negative = 0.
**Body Mass Index (BMI)** *(ANU-ADRI, CAIDE, CogDrisk, LIBRA, LIBRA2, Lancet)*	HUNT3: Bmi_NT3BLM	On the HUNT3 visit day, weight was measured to the nearest 0.1 kg with participants wearing light clothing and no shoes, and height to the nearest 0.01 m using a wall-mounted stadiometer. BMI was calculated as kg/m² and grouped into underweight (< 18.5), normal (18.5–24.99), overweight (25–29.99), and obese (≥ 30)[2].
**Cognitive activity** *(ANU-ADRI, CogDrisk; LIBRA)*	HUNT3: ActPCWorHD_NT3BLQ2, ActPCLeiHD_NT3BLQ2, CulExhL6M_NT3BLQ2, CulConceL6M_NT3BLQ2, ActMusL6M_NT3BLQ2; composites SumCogActScore_w3, CogActScore_w3	Five items were selected for conceptual overlap with ANU-ADRI and CogDrisk: computer use at work, computer use for leisure, visits to museums/exhibitions, attendance at concerts/theatre/film, and participation in music/theatre activities. Each item was scored 0 (never), 1 (≤ monthly), 2 (weekly), or 3 (daily/almost daily). Scores were summed (range 0–15) and split into terciles yielding low, medium and high cognitive activity.
**Depression** *(ANU-ADRI, CogDrisk, LIBRA, LIBRA2, UKBDRS, Lancet)*	HUNT3: HADSDepr_NT3BLQ2; NPR (ICD10): F32–F33	Participant with a score ≥ 8 on the Hospital Anxiety and Depression Scale[3] or had an ICD-10 diagnosis of depressive episode/recurrent depression (F32–F33) in the Norwegian Patient Register between 2007 and 2009 were coded as depressed.
**Diabetes** *(ANU-ADRI, CAIDE, CogDrisk, LIBRA, LIBRA2, UKBDRS, Lancet)*	HUNT1: DiaEv_NT1BLQ1, BloGluNonFast_NT1BLM; HUNT 2: DiaEv_NT2BLQ1, SeGluNonFast_NT2BLM; HUNT3: DiaEv_NT3Dia2Q, SeGluNonFast_NT3BLM; NPR (ICD10): E10–E14	Diabetes was coded “yes” if the participant self-reported diabetes or had non-fasting glucose ≥ 9 mmol/L[4] at HUNT3 or earlier HUNT waves, or NPR carried an ICD-10 code E10–E14.

**Education** *(ANU-ADRI, CAIDE, CogDrisk, UKBDRS, Lancet)*	Statistics Norway (SSB): education register 2007: EDU_2007	SSB variable EDU_2007International Standard Classification of Education (ISCED) values < 200 000 were coded primary (< 8 y; education_w3 = 0), 200 000–399 999 secondary (8–12 y; = 1), and ≥ 400 000 tertiary (≥ 12 y; = 2). For UKBDRS continuous years of education were coded by mapping SSB codes to mid-point year values (e.g., 200 000→7 y, 230 000→12 y, 310 000→17 y).
**Fish consumption** *(ANU-ADRI; CogDrisk)*	HUNT3: FoFishF_NT3BLQ1	Self-reported consumption of fish was categorised into 0–3 times/month, 1–3 times/week, 4–6 times/week, or ≥ 1 time/day.
**Healthy diet (Mediterranean-style proxy)** *(LIBRA; LIBRA2)*	HUNT3: FoFruF_NT3BLQ1, FoVegF_NT3BLQ1, FoFishF_NT3BLQ1	Fruit, vegetable and fruit consumption was coded 1 point for 0–3 times/month, 2 for 1–3 times/week, 3 for 4–6 times/week, and 4 for daily or more. Points were summed (range 3–12). Scores ≥ 10 defined a "healthy diet".
**Hearing loss** *(LIBRA2, Lancet)*	HUNT1: HearImp_NT1BLQ1; HUNT2: HearLosCu_NT2HearQ1, HearImp_NT2HearQ2	Participants were asked whether they were aware of impaired hearing (yes/no) at each HUNT wave. A single positive response in any wave permanently classified the individual as having hearing loss.
**Heart disease** *(LIBRA risk factor; CogDrisk uses AF; UKBDRS has cardiovascular history items)*	HUNT3: CarAngEv_NT3BLQ1/NP, MI CarInfEv_NT3BLQ1/NP, CarFaiEv_NT3BLQ1, CarDisOtEv_NT3BLQ1; NPR (ICD10): I11, I20–I25, I50–I51	To approximate coronary heart disease/atrial fibrillation across indices, we created a proxy variable: participants self-reporting physician-diagnosed angina, myocardial infarction, heart failure, or other specified heart disease, or carrying any corresponding NPR code (ICD10: I11, I20-25, I50-51), were coded "heart disease = yes".
**Hypertension** *(CAIDE, CogDrisk, LIBRA, LIBRA2, UKBDRS, Lancet)*	HUNT3: BPSystMn23_NT3BLM; NPR (ICD10): I10, I15	Blood pressure was measured after 2 min seated rest, three times at 1-min intervals. The mean of the second and third readings was used. Hypertension was defined as SBP ≥ 140 mm Hg, or an NPR diagnosis I10/I15.
**Lives alone** *(UKBDRS)*	SSB Household register: pers_i_hushnr; HUNT3: CohSps_NT3BLQ2,	Household composition data from the National Population Register was used in combination with self-reported living status. Participants registered as the sole resident in their dwelling were coded "lives alone = yes"
**Material deprivation / SES** *(UKBDRS)*	SSB: net incom, net assets	SSB annual net income and household assets were averaged across available tax years (2007-2009). Low SES was flagged when income or wealth fell in the bottom quintile. A composite "material deprivation" variable equalled 1 when low-income was present and asset quintile < 5.
**Parental dementia**	HUNT4 70+: DiagDemFam1_NT4EldRelal, DiagDemFam1_NT4Eld	At HUNT4 70+, participants self-reported whether either parent had had a history of dementia.
**Physical activity** *(ANU-ADRI, CAIDE, CogDrisk, LIBRA, LIBRA2, Lancet)*	HUNT3: *ExeF_NT3BLQ1, *ExeLigDuLY_NT3BLQ2, ExeHarDuLY_NT3BLQ2	Weekly minutes of moderate and vigorous activity were calculated based on self-reported HUNT3 exercise frequency. Moderate activity was assigned 0, 30, 90, or 180 minutes per week, and vigorous activity 0, 60, 180, or 360 minutes per week. These were summed to total moderate-to-vigorous physical activity (MVPA). Participants were classified as low (≤29 min/week), medium (30–149 min/week), or high (≥150 min/week).
**Sleep disturbance** *(LIBRA2; CogDrisk)*	HUNT3: InsomEvnL3M_NT3BLQ2, InsomNigL3M_NT3BLQ2, InsomMornL3M_NT3BLQ2	Participants who reported experiencing difficulties falling asleep, nocturnal awakenings, or early-morning awakenings "several times a week" on any of the three items were coded as having sleep disturbance.
**Smoking status** *(ANU-ADRI, CogDrisk, LIBRA, LIBRA2, Lancet)*	HUNT3: SmoStat_NT3BLQ1	Participants self-reported reported current, former, or never smoking and were coded: (i) current smoker vs non-current (former/never) for indices that use a binary risk score; and (ii) current, ex-, never for ANU-ADRI, which assigns intermediate risk to ex-smokers.
**Social activity / engagement** *(ANU-ADRI social engagement; LIBRA low social activity; CogDrisk loneliness; Lancet social isolation)*	HUNT3: *CohSpsNT3BLQ2, NwFNwUnite/NwDistru/NwWelf_NT3BLQ2, ActOrgL6M/ActChuL6M_NT3BLQ2, FeelLoneL2W_NT3BLQ1lf_NT3BLQ2, ActOrgL6M/ActChuL6M_NT3BLQ2, FeelLoneL2W_NT3BLQ1	Social activity was assessed using multiple self-reported measures, selected to imitate the ANU-ADRI survey as closely as HUNT3 data allowed. ANU-ADRI considers five factors: (1) marital status, (2) social network size, (3) social network quality, (4) level of social activities, and (5) living arrangements. In HUNT3, these were collapsed into three domains: (1) marital/cohabitation status, (2) social-network quality, and (3) social-activity level.<br>Marital/cohabitation: participants living with a spouse or partner were assigned 4 points; others 0 points.<br>Social-network quality: five items captured perceived support, community, trust, and cohesion (e.g., "Has friends who provide help," "Has friends to talk to confidentially," "Sense of community," "Trust in people," "People like living here"). Responses were rescaled and summed to a maximum of 4 points, with higher values indicating better quality.

		Social-activity level: participation in associations and church activities over the past six months was coded by frequency (never to very frequent) and summed to a maximum of 4 points.<br>Domain scores were summed into a total social activity score (0–12). Participants were divided into quartiles, with the lowest quartile classified as "low social activity" for LIBRA scoring. For CogDrisk and the Lancet model, social engagement was instead derived from the loneliness item: participants reporting "some" or "strong" loneliness were coded as lonely, and those reporting "not at all" or "a little" as not lonely.
**Stroke** *(CogDrisk; UKBDRS)*	HUNT1: ApoplEv_NT1BLQ1; HUNT2: ApoplEv_NT2BLQ1; HUNT3: ApoplEv_NT3BLQ1 ; NPR (ICD10) I60–I69	Stroke history was positive if the participant self-reported a stroke in HUNT3 or had an NPR code I60–I69 (sub-arachnoid, intracerebral, ischaemic, or unspecified stroke) between 2007 and 2009. Earlier HUNT waves were carried over if participants indicated a stroke history at HUNT1 or 2.
**Total cholesterol** *(ANU-ADRI, LIBRA, LIBRA2; CAIDE, UKBDRS, Lancet)*	HUNT3: SeChol_NT3BLM	Total cholesterol was measured enzymatically from non-fasting venous blood. Following cut-off values established in CAIDE. < 6.5 mmol/L were coded as "normal" and ≥ 6.5 mmol/L as "high"[5].
**Traumatic brain injury (TBI)** *(ANU-ADRI, CogDrisk, Lancet)*	HUNT2: HospHeadInju_NT2HearQ1; NPR S06–S07, S09	TBI was considered present if the participant reported a hospitalised head injury at HUNT2 or had an NPR code indicating intracranial injury (S06–S07) or other head injury (S09) during 2007–2009.
**Dementia Diagnosis**	HUNT4 70+	Dementia diagnoses in HUNT4 70+ were established by a Diagnostic Consensus Committee of nine physicians with expertise in geriatrics, neurology, and old-age psychiatry, combining clinical and research experience[6]. Diagnoses were made according to the Diagnostic and Statistical Manual of Mental Disorders, Fifth Edition (DSM-5), and participants were classified into four categories: (1) no cognitive impairment, (2) amnestic mild cognitive impairment, (3) non-amnestic mild cognitive impairment, and (4) dementia. Dementia cases were further subtyped into Alzheimer's disease (AD) and other dementias. For this study, we coded dementia as a binary variable: "No Dementia" (no or mild cognitive impairment) and "Dementia" (any dementia diagnosis). Dementia free participants were included in the analysis.
Variables not available in HUNT (vision loss, air pollution, chronic kidney disease, pesticide exposure) were excluded.		

RESULTS

Risk Scores Predicting Cognitive Decline (Continous)

Main Results Full Table

Supplementary Table 3) Mixed Effects Models of Continous (z-score) Risk Scores With, and Without Demographics - With IPW

Term	ANU-ADRI β [95%CI]	CAIDE β [95%CI]	CogDrisk β [95%CI]	LIBRA β [95%CI]	LIBRA2 β [95%CI]	UKBDRS β [95%CI]	UKBDRS-APOE β [95%CI]	Lancet β [95%CI]
**Continuous Risk Scores (z-score) With Demographics**								
**Intercept**	23.11*** [23.02, 23.21]	23.68*** [23.60, 23.76]	23.15*** [23.06, 23.23]	23.11*** [23.02, 23.20]	23.47*** [23.38, 23.55]	23.22*** [23.13, 23.31]	23.24*** [23.15, 23.33]	23.19*** [23.10, 23.29]
**Time (per year)**	-0.40*** [-0.43, -0.36]	-0.30*** [-0.33, -0.28]	-0.38*** [-0.41, -0.35]	-0.39*** [-0.42, -0.35]	-0.32*** [-0.35, -0.30]	-0.41*** [-0.44, -0.37]	-0.41*** [-0.44, -0.38]	-0.42*** [-0.45, -0.38]
**Risk Score (z)**	-1.57*** [-1.67, -1.46]	-0.74*** [-0.82, -0.67]	-1.53*** [-1.63, -1.44]	-1.58*** [-1.68, -1.48]	-0.97*** [-1.06, -0.89]	-1.61*** [-1.72, -1.51]	-1.51*** [-1.62, -1.41]	-1.50*** [-1.62, -1.39]
**Time × Score (z)**	-0.17*** [-0.21, -0.13]	-0.04*** [-0.07, -0.02]	-0.15*** [-0.19, -0.12]	-0.16*** [-0.19, -0.12]	-0.07*** [-0.09, -0.04]	-0.22*** [-0.26, -0.18]	-0.22*** [-0.26, -0.19]	-0.23*** [-0.27, -0.18]
**Variance (Intercept)**	5.70	6.57	8.58	6.40	6.56	5.77	8.03	6.18
**Variance (Time)**	0.37	0.34	0.23	0.31	0.33	0.35	0.24	0.34
**AIC**	-197536.66	-196040.05	-194087.62	-196819.18	-196159.18	-197557.88	-194773.14	-196888.91
**Continuous Risk Scores (z-score) Without Demographics**								
**Term**	**ANU-ADRI β [95%CI]**	**CAIDE β [95%CI]**	**CogDrisk β [95%CI]**	**LIBRA β [95%CI]**	**LIBRA2 β [95%CI]**	**UKBDRS β [95%CI]**	**UKBDRS-APOE β [95%CI]**	**Lancet β [95%CI]**
**Intercept**	23.73 [23.65, 23.81]***	23.80 [23.73, 23.88]***	23.70 [23.62, 23.78]***	23.66 [23.58, 23.74]***	23.67 [23.59, 23.75]***	23.82 [23.74, 23.90]***	23.82 [23.75, 23.90]***	23.72 [23.64, 23.80]***
**Time (per year)**	-0.30 [-0.32, -0.27]***	-0.29 [-0.32, -0.27]***	-0.30 [-0.33, -0.28]***	-0.30 [-0.33, -0.28]***	-0.30 [-0.33, -0.30]***	-0.30 [-0.32, -0.28]***	-0.30 [-0.32, -0.27]***	-0.30 [-0.33, -0.28]***
**Risk Score (z)**	-0.47 [-0.55, -0.38]***	-0.33 [-0.41, -0.25]***	-0.56 [-0.64, -0.49]***	-0.64 [-0.72, -0.57]***	-0.63 [-0.71, -0.55]***	-0.34 [-0.43, -0.26]***	-0.25 [-0.33, -0.17]***	-0.50 [-0.58, -0.41]***
**Time × Score (z)**	-0.02 [-0.05, 0.00]	-0.03 [-0.06, -0.01]***	-0.04 [-0.06, -0.02]***	-0.03 [-0.05, -0.01]***	-0.03 [-0.06, -0.01]***	-0.06 [-0.09, -0.04]***	-0.07 [-0.09, -0.05]***	-0.04 [-0.07, -0.02]***
**Variance (intercept)**	6.99	7.97	5.86	4.96	5.25	5.27	8.10	7.34
**Variance (time)**	0.33	0.28	0.43	0.66	0.56	0.59	0.28	0.30
**AIC**	-195353.84	-194227.13	-196495.18	-196800.73	-196803.91	-196474.65	-194094.52	-194986.60

Notes: * p≤0.05; ** p≤0.01, *** p≤0.001

Sensitivity Analysis

Supplementary Table 4) Mixed Effects Models of Continuous (z-score) Risk Scores With, and Without Demographics - No Inverse Probability Weights

Continuous Risk Scores (z-score) With Demographics

Term	ANU-ADRI β [95%CI]	CAIDE β [95%CI]	CogDrisk β [95%CI]	LIBRA β [95%CI]	LIBRA2 β [95%CI]	UKBDRS β [95%CI]	UKBDRS-APOE β [95%CI]	LANCET β [95%CI]
**Intercept**	23.11*** [23.02, 23.20]	23.68*** [23.60, 23.75]	23.14*** [23.06, 23.23]	23.10*** [23.01, 23.19]	23.46*** [23.38, 23.55]	23.22*** [23.13, 23.30]	23.24*** [23.15, 23.32]	23.19*** [23.10, 23.28]
**Time (per year)**	-0.36*** [-0.40, -0.33]	-0.27*** [-0.29, -0.25]	-0.35*** [-0.38, -0.32]	-0.36*** [-0.39, -0.32]	-0.29*** [-0.32, -0.26]	-0.37*** [-0.40, -0.34]	-0.37*** [-0.40, -0.34]	-0.38*** [-0.42, -0.34]
**Risk index (z)**	-1.57*** [-1.68, -1.47]	-0.75*** [-0.82, -0.67]	-1.54*** [-1.64, -1.44]	-1.58*** [-1.68, -1.48]	-0.98*** [-1.07, -0.89]	-1.62*** [-1.72, -1.51]	-1.52*** [-1.62, -1.41]	-1.51*** [-1.63, -1.39]
**Time × index (z)**	-0.16*** [-0.20, -0.12]	-0.04*** [-0.07, -0.02]	-0.14*** [-0.17, -0.11]	-0.15*** [-0.18, -0.11]	-0.06*** [-0.09, -0.04]	-0.20*** [-0.24, -0.16]	-0.20*** [-0.23, -0.16]	-0.21*** [-0.25, -0.16]
**Var(Intercept)**	6.39	7.20	6.22	6.21	7.04	6.32	6.40	6.67
**Var(Time)**	0.22	0.25	0.22	0.22	0.25	0.21	0.21	0.23
**AIC**	58 274.05	58 945.60	58 159.81	58 159.12	58 802.43	58 161.44	58 220.40	58 425.22

Continuous Risk Scores (z-score) Without Demographics

Term	ANU-ADRI β [95%CI]	CAIDE β [95%CI]	CogDrisk β [95%CI]	LIBRA β [95%CI]	LIBRA2 β [95%CI]	UKBDRS β [95%CI]	UKBDRS-APOE β [95%CI]	LANCET β [95%CI]
**Intercept**	23.72*** [23.64, 23.80]	23.80*** [23.72, 23.87]	23.70*** [23.62, 23.78]	23.65*** [23.58, 23.73]	23.66*** [23.58, 23.74]	23.81*** [23.74, 23.89]	23.82*** [23.74, 23.90]	23.72*** [23.64, 23.80]
**Time (per year)**	-0.26*** [-0.29, -0.24]	-0.26*** [-0.28, -0.24]	-0.27*** [-0.30, -0.25]	-0.27*** [-0.29, -0.25]	-0.27*** [-0.29, -0.25]	-0.26*** [-0.29, -0.24]	-0.27*** [-0.29, -0.24]	-0.27*** [-0.29, -0.25]
**Risk index (z)**	-0.47*** [-0.56, -0.39]	-0.33*** [-0.41, -0.25]	-0.57*** [-0.65, -0.49]	-0.65*** [-0.73, -0.57]	-0.64*** [-0.72, -0.55]	-0.34*** [-0.43, -0.25]	-0.25*** [-0.33, -0.17]	-0.50*** [-0.59, -0.41]
**Time × index (z)**	-0.02 [-0.04, 0.00]	-0.04** [-0.06, -0.01]	-0.04*** [-0.06, -0.02]	-0.03** [-0.05, -0.01]	-0.03** [-0.06, -0.01]	-0.06*** [-0.08, -0.03]	-0.07*** [-0.09, -0.04]	-0.04** [-0.07, -0.02]
**Var(intercept)**	7.69	7.76	7.52	7.41	7.47	7.79	7.81	7.65
**Var(time)**	0.26	0.26	0.26	0.26	0.26	0.26	0.26	0.26
**AIC**	59 248.18	59 297.06	59 148.21	59 074.59	59 114.99	59 293.99	59 305.48	59 220.82

Notes: * p≤0.05; ** p≤0.01, *** p≤0.001

Supplementary Table 5) Mixed Effects Models of Continuous (z-score) Risk Scores With, and Without Demographics - No Inverse Probability Weights, complete cases only

Continuous Risk Scores (z-score) With Demographics

Term	ANU-ADRI β [95%CI]	CAIDE β [95%CI]	CogDrisk β [95%CI]	LIBRA β [95%CI]	LIBRA2 β [95%CI]	UKBDRS β [95%CI]	UKBDRS-APOE β [95%CI]	LANCET β [95%CI]
**Intercept**	23.64*** [23.51, 23.76]	24.22*** [24.13, 24.31]	23.67*** [23.55, 23.79]	23.59*** [23.47, 23.71]	24.01*** [23.91, 24.11]	23.79*** [23.67, 23.91]	23.84*** [23.72, 23.96]	23.80*** [23.67, 23.93]
**Time (per year)**	-0.40*** [-0.44, -0.37]	-0.31*** [-0.33, -0.28]	-0.39*** [-0.42, -0.36]	-0.39*** [-0.43, -0.36]	-0.33*** [-0.36, -0.30]	-0.41*** [-0.45, -0.38]	-0.42*** [-0.45, -0.38]	-0.43*** [-0.46, -0.39]
**Risk index (z)**	-1.31*** [-1.45, -1.17]	-0.71*** [-0.79, -0.63]	-1.27*** [-1.39, -1.15]	-1.36*** [-1.49, -1.24]	-0.84*** [-0.94, -0.74]	-1.23*** [-1.38, -1.09]	-1.13*** [-1.26, -0.99]	-1.15*** [-1.31, -0.98]
**Time × index (z)**	-0.18*** [-0.22, -0.14]	-0.05*** [-0.07, -0.02]	-0.16*** [-0.20, -0.13]	-0.16*** [-0.20, -0.13]	-0.07*** [-0.10, -0.05]	-0.23*** [-0.27, -0.19]	-0.23*** [-0.27, -0.19]	-0.24*** [-0.28, -0.19]
**Var(Intercept)**	5.62	5.98	5.45	5.42	5.94	5.63	5.67	5.89
**Var(Time)**	0.26	0.29	0.25	0.25	0.29	0.24	0.24	0.26
**AIC**	45020.03	45263.91	44927.77	44915.67	45239.36	44985.52	44992.04	45135.91

Continuous Risk Scores (z-score) Without Demographics

Term	ANU-ADRI β [95%CI]	CAIDE β [95%CI]	CogDrisk β [95%CI]	LIBRA β [95%CI]	LIBRA2 β [95%CI]	UKBDRS β [95%CI]	UKBDRS-APOE β [95%CI]	LANCET β [95%CI]
**Intercept**	24.28*** [24.19, 24.37]	24.38*** [24.29, 24.47]	24.25*** [24.16, 24.34]	24.19*** [24.09, 24.28]	24.21*** [24.12, 24.30]	24.40*** [24.32, 24.49]	24.41*** [24.32, 24.50]	24.29*** [24.20, 24.38]
**Time (per year)**	-0.30*** [-0.32, -0.28]	-0.30*** [-0.32, -0.28]	-0.31*** [-0.33, -0.29]	-0.31*** [-0.33, -0.28	-0.31*** [-0.33, -0.28	-0.30*** [-0.32, -0.28	-0.30*** [-0.32, -0.28]	-0.31*** [-0.33, -0.28]
**Risk index (z)**	-0.46*** [-0.56, -0.37]	-0.35*** [-0.43, -0.26]	-0.53*** [-0.62, -0.44	-0.62*** [-0.71, -0.53	-0.61*** [-0.70, -0.52	-0.25*** [-0.36, -0.14	-0.20*** [-0.30, -0.11]	-0.45*** [-0.55, -0.35]
**Time × index (z)**	-0.02 [-0.05, 0.00]	-0.04** [-0.06, -0.01]	-0.04*** [-0.07, -0.02]	-0.03** [-0.05, -0.01]	-0.04** [-0.06, -0.01]	-0.06*** [-0.09, -0.04]	-0.07*** [-0.09, -0.05	-0.04*** [-0.07, -0.02

**Var(intercept)**	6.40	6.45	6.27	6.16	6.21	6.52	6.53	6.39
**Var(time)**	0.31	0.30	0.30	0.30	0.30	0.30	0.30	0.30
**AIC**	45489.06	45514.53	45419.96	45360.48	45389.66	45538.54	45529.99	45480.16

Notes: * p≤0.05; ** p≤0.01, *** p≤0.001

Predicting Cognitive Decline by Risk Group

Main Results Full Table

Supplementary Table 5) Mixed models of Risk Score Terciles With, and Without Demographics

Risk Score Terciles Without Demographics

	**ANU-ADRI β [95%CI]**	**CAIDE β [95%CI]**	**CogDrisk β [95%CI]**	**LIBRA β [95%CI]**	**LIBRA2 β [95%CI]**	**UKBDRS β [95%CI]**	**UKBDRS-APOE β [95%CI]**	**Lancet β [95%CI]**
**Intercept**	24.62*** [24.53, 24.72]	24.50*** [24.40, 24.61]	24.76*** [24.66, 24.85]	24.75*** [24.65, 24.84]	24.52*** [24.42, 24.63]	24.74*** [24.63, 24.84]	24.72*** [24.62, 24.82]	24.54*** [24.44, 24.65]
**Time (per year)**	-0.23*** [-0.26, -0.20]	-0.25*** [-0.28, -0.22]	-0.21*** [-0.24, -0.19]	-0.23*** [-0.25, -0.20]	-0.25*** [-0.27, -0.22]	-0.19*** [-0.22, -0.16]	-0.19*** [-0.21, -0.16]	-0.20*** [-0.22, -0.17]
**Medium Risk (vs Low)**	-1.20*** [-1.36, -1.04]	-1.00*** [-1.16, -0.83]	-1.31*** [-1.47, -1.16]	-1.30*** [-1.46, -1.15]	-1.00*** [-1.16, -0.83]	-1.08*** [-1.24, -0.93]	-1.08*** [-1.23, -0.92]	-0.83*** [-0.98, -0.67]
**High Risk (vs Low)**	-2.94*** [-3.19, -2.68]	-1.65*** [-1.86, -1.44]	-3.04*** [-3.27, -2.81]	-3.04*** [-3.27, -2.80]	-1.86*** [-2.08, -1.65]	-3.02*** [-3.25, -2.79]	-3.00*** [-3.24, -2.77]	-2.61*** [-2.85, -2.37]
**Time × Medium Risk**	-0.11*** [-0.16, -0.06]	-0.09*** [-0.14, -0.04]	-0.12*** [-0.17, -0.08]	-0.09*** [-0.13, -0.04]	-0.06* [-0.11, -0.01]	-0.16*** [-0.20, -0.11]	-0.16*** [-0.20, -0.11]	-0.15*** [-0.20, -0.11]
**Time × High Risk**	-0.32*** [-0.43, -0.22]	-0.07* [-0.13, 0.00]	-0.35*** [-0.44, -0.26]	-0.38*** [-0.47, -0.28]	-0.15*** [-0.22, -0.08]	-0.40*** [-0.49, -0.31]	-0.43*** [-0.52, -0.34]	-0.39*** [-0.48, -0.30]
**Variance (Intercept)**	4.93	5.20	6.13	6.21	5.58	7.28	7.76	5.34
**Variance (Time)**	0.55	0.56	0.34	0.33	0.45	0.27	0.26	0.46
AIC	-197111.43	-196313.82	-196397.11	-196304.30	-196321.49	-195005.84	-194422.63	-196806.06

Risk Score Terciles Without Demographics

	**ANU-ADRI β [95%CI]**	**CAIDE β [95%CI]**	**CogDrisk β [95%CI]**	**LIBRA β [95%CI]**	**LIBRA2 β [95%CI]**	**UKBDRS β [95%CI]**	**UKBDRS-APOE β [95%CI]**	**Lancet β [95%CI]**
**Intercept**	24.21*** [24.10, 24.31]	24.06*** [23.95, 24.16]	24.31*** [24.20, 24.43]	24.37*** [24.26, 24.48]	24.38*** [24.27, 24.49]	24.10*** [23.99, 24.21]	24.12*** [24.00, 24.25]	24.23*** [24.12, 24.34]
**Time (per year)**	-0.28*** [-0.31, -0.25]	-0.25*** [-0.28, -0.23]	-0.24*** [-0.27, -0.21]	-0.27*** [-0.30, -0.24]	-0.26*** [-0.29, -0.23]	-0.25*** [-0.28, -0.22]	-0.22*** [-0.25, -0.19]	-0.27*** [-0.30, -0.24]
**Medium Risk (vs Low)**	-0.60*** [-0.78, -0.42]	-0.16 [-0.35, 0.04]	-0.63*** [-0.80, -0.47]	-0.73*** [-0.90, -0.56]	-0.67*** [-0.84, -0.50]	-0.32** [-0.53, -0.12]	-0.41*** [-0.59, -0.23]	-0.45*** [-0.63, -0.27]
**High Risk (vs Low)**	-0.90*** [-1.09, -0.70]	-0.69*** [-0.88, -0.51]	-1.15*** [-1.36, -0.95]	-1.36*** [-1.56, -1.16]	-1.35*** [-1.54, -1.15]	-0.61*** [-0.78, -0.43]	-0.49*** [-0.67, -0.30]	-1.05*** [-1.25, -0.86]

**Time × Medium Risk**	0.02 [-0.04, 0.07]	-0.08** [-0.13, -0.02]	-0.07** [-0.12, -0.02]	-0.03 [-0.08, 0.02]	-0.05 [-0.10, 0.00]	-0.09** [-0.15, -0.03]	-0.07** [-0.12, -0.02]	-0.00 [-0.05, 0.05]
**Time × High Risk**	-0.03 [-0.09, 0.03]	-0.06* [-0.12, -0.01]	-0.10** [-0.16, -0.04]	-0.05 [-0.11, 0.01]	-0.04 [-0.10, 0.01]	-0.08** [-0.13, -0.03]	-0.15*** [-0.20, -0.10]	-0.10** [-0.15, -0.04]
**Variance (Intercept)**	9.40	6.85	7.19	6.38	5.26	4.98	9.50	5.35
**Variance (Time)**	0.24	0.34	0.31	0.37	0.57	0.74	0.24	0.55
**AIC**	-192077.04	-194856.21	-194566.82	-195458.07	-196127.12	-195642.71	-191969.57	-196584.64

Notes: All baseline participants (n=7221) and follow up participants (n=4716) are included; * p≤0.05; ** p≤0.01, *** p≤0.001)

Sensitivity Analysis

Supplementary Table 6) Mixed models of Risk Score Terciles With, and Without Demographics - No Inverse Probability Weights

Risk Score Terciles Without Demographics

	**ANU-ADRI β [95%CI]**	**CAIDE β [95%CI]**	**CogDrisk β [95%CI]**	**LIBRA β [95%CI]**	**LIBRA2 β [95%CI]**	**UKBDRS β [95%CI]**	**UKBDRS-APOE β [95%CI]**	**LANCET β [95%CI]**
**Intercept**	24.62*** [24.53, 24.72]	24.50*** [24.40, 24.61]	24.76*** [24.66, 24.85]	24.75*** [24.65, 24.84]	24.52*** [24.42, 24.63]	24.74*** [24.63, 24.84]	24.72*** [24.62, 24.82]	24.54*** [24.44, 24.65]
**Time (per year)**	-0.23*** [-0.26, -0.20]	-0.25*** [-0.28, -0.22]	-0.21*** [-0.24, -0.19]	-0.23*** [-0.25, -0.20]	-0.25*** [-0.27, -0.22]	-0.19*** [-0.22, -0.16]	-0.19*** [-0.21, -0.16]	-0.20*** [-0.22, -0.17]
**Medium Risk (vs Low)**	-1.20*** [-1.36, -1.04]	-1.00*** [-1.16, -0.83]	-1.31*** [-1.47, -1.16]	-1.30*** [-1.46, -1.15]	-1.00*** [-1.16, -0.83]	-1.08*** [-1.24, -0.93]	-1.08*** [-1.23, -0.92]	-0.83*** [-0.98, -0.67]
**High Risk (vs Low)**	-2.94*** [-3.19, -2.68]	-1.65*** [-1.86, -1.44]	-3.04*** [-3.27, -2.81]	-3.04*** [-3.27, -2.80]	-1.86*** [-2.08, -1.65]	-3.02*** [-3.25, -2.79]	-3.00*** [-3.24, -2.77]	-2.61*** [-2.85, -2.37]
**Time × Medium Risk**	-0.11*** [-0.16, -0.06]	-0.09*** [-0.14, -0.04]	-0.12*** [-0.17, -0.08]	-0.09*** [-0.13, -0.04]	-0.06* [-0.11, -0.01]	-0.16*** [-0.20, -0.11]	-0.16*** [-0.20, -0.11]	-0.15*** [-0.20, -0.11]
**Time × High Risk**	-0.32*** [-0.43, -0.22]	-0.07* [-0.13, 0.00]	-0.35*** [-0.44, -0.26]	-0.38*** [-0.47, -0.28]	-0.15*** [-0.22, -0.08]	-0.40*** [-0.49, -0.31]	-0.43*** [-0.52, -0.34]	-0.39*** [-0.48, -0.30]
**Var(Intercept)**	4.93	5.20	6.13	6.21	5.58	7.28	7.76	5.34
**Var(Time)**	0.55	0.56	0.34	0.33	0.45	0.27	0.26	0.46
**AIC**	-197111.43	-196313.82	-196397.11	-196304.30	-196321.49	-195005.84	-194422.63	-196806.06

Risk Score Terciles Without Demographics

	**ANU-ADRI β [95%CI]**	**CAIDE β [95%CI]**	**CogDrisk β [95%CI]**	**LIBRA β [95%CI]**	**LIBRA2 β [95%CI]**	**UKBDRS β [95%CI]**	**UKBDRS-APOE β [95%CI]**	**LANCET β [95%CI]**
**Intercept**	24.21*** [24.10, 24.31]	24.05*** [23.95, 24.16]	24.31*** [24.20, 24.43]	24.37*** [24.26, 24.48]	24.37*** [24.26, 24.48]	24.10*** [23.99, 24.20]	24.12*** [24.00, 24.25]	24.23*** [24.12, 24.34]
**Time (per year)**	-0.26*** [-0.29, -0.23]	-0.22*** [-0.25, -0.19]	-0.22*** [-0.25, -0.18]	-0.24*** [-0.27, -0.21]	-0.24*** [-0.27, -0.21]	-0.22*** [-0.25, -0.19]	-0.20*** [-0.23, -0.16]	-0.24*** [-0.27, -0.21]
**Medium Risk (vs Low)**	-0.60*** [-0.78, -0.42]	-0.16 [-0.35, 0.04]	-0.63*** [-0.80, -0.46]	-0.73*** [-0.90, -0.56]	-0.67*** [-0.84, -0.50]	-0.32** [-0.52, -0.12]	-0.41*** [-0.60, -0.23]	-0.46*** [-0.64, -0.28]
**High Risk (vs Low)**	-0.90*** [-1.10, -0.71]	-0.70*** [-0.88, -0.51]	-1.16*** [-1.37, -0.96]	-1.37*** [-1.57, -1.17]	-1.35*** [-1.55, -1.16]	-0.61*** [-0.78, -0.43]	-0.48*** [-0.67, -0.30]	-1.06*** [-1.25, -0.87]
**Time × Medium Risk**	0.02 [-0.04, 0.07]	-0.08** [-0.14, -0.02]	-0.07** [-0.12, -0.02]	-0.03 [-0.08, 0.02]	-0.05 [-0.10, 0.00]	-0.08** [-0.14, -0.02]	-0.06* [-0.11, -0.01]	0.00 [-0.05, 0.05]
**Time × High Risk**	-0.02 [-0.08, 0.04]	-0.07** [-0.12, -0.02]	-0.09** [-0.16, -0.03]	-0.05 [-0.11, 0.01]	-0.04 [-0.10, 0.02]	-0.08** [-0.13, -0.03]	-0.14*** [-0.19, -0.09]	-0.09** [-0.15, -0.03]
**Var(Intercept)**	7.77	7.79	7.64	7.58	7.59	7.83	7.84	7.68
**Var(Time)**	0.27	0.26	0.26	0.26	0.26	0.26	0.26	0.26
**AIC**	59285.66	59314.12	59217.37	59172.53	59174.72	59320.23	59318.38	59240.55

Notes: All baseline participants (n=7221) and follow up participants (n=4716) are included; * p≤0.05; ** p≤0.01, *** p≤0.001)

Supplementary Table 7) Mixed models of Risk Score Terciles With, and Without Demographics - Complete Cases Only

Risk Score Terciles Without Demographics								
	**ANU-ADRI β [95%CI]**	**CAIDE β [95%CI]**	**CogDrisk β [95%CI]**	**LIBRA β [95%CI]**	**LIBRA2 β [95%CI]**	**UKBDRS β [95%CI]**	**UKBDRS-APOE β [95%CI]**	**LANCET β [95%CI]**
**Intercept**	24.95*** [24.85, 25.06]	25.00*** [24.88, 25.12]	25.06*** [24.95, 25.16]	25.05*** [24.94, 25.15]	24.95*** [24.83, 25.06]	24.98*** [24.86, 25.09]	24.98*** [24.87, 25.09]	24.84*** [24.73, 24.95]
**Time (per year)**	-0.23*** [-0.26, -0.21]	-0.25*** [-0.28, -0.22]	-0.22*** [-0.24, -0.19]	-0.23*** [-0.26, -0.20]	-0.25*** [-0.28, -0.22]	-0.19*** [-0.22, -0.16]	-0.19*** [-0.22, -0.16]	-0.20*** [-0.23, -0.17]
**Medium Risk (vs Low)**	-1.15*** [-1.34, -0.96]	-0.85*** [-1.04, -0.67]	-1.19*** [-1.38, -1.01]	-1.20*** [-1.38, -1.02]	-0.90*** [-1.09, -0.71]	-0.89*** [-1.07, -0.71]	-0.88*** [-1.06, -0.70]	-0.65*** [-0.83, -0.47]
**High Risk (vs Low)**	-2.11*** [-2.48, -1.75]	-1.67*** [-1.92, -1.42]	-2.36*** [-2.68, -2.04]	-2.37*** [-2.70, -2.04]	-1.55*** [-1.81, -1.30]	-2.05*** [-2.37, -1.72]	-2.11*** [-2.43, -1.78]	-1.76*** [-2.10, -1.43]
**Time × Medium Risk**	-0.11*** [-0.16, -0.06]	-0.09*** [-0.14, -0.04]	-0.13*** [-0.18, -0.08]	-0.09*** [-0.14, -0.04]	-0.07** [-0.11, -0.02]	-0.16*** [-0.21, -0.12]	-0.16*** [-0.21, -0.12]	-0.16*** [-0.20, -0.11]
**Time × High Risk**	-0.35*** [-0.45, -0.25]	-0.07* [-0.14, -0.01]	-0.37*** [-0.46, -0.29]	-0.40*** [-0.49, -0.31]	-0.16*** [-0.23, -0.09]	-0.42*** [-0.51, -0.33]	-0.45*** [-0.54, -0.36]	-0.41*** [-0.50, -0.32]
**Var(Intercept)**	5.88	6.14	5.66	5.69	6.13	5.89	5.83	6.07
**Var(Time)**	0.27	0.30	0.26	0.26	0.29	0.26	0.25	0.27
**AIC**	45180.03	45345.54	45043.19	45050.80	45338.60	45139.85	45097.06	45236.95

Risk Score Terciles Without Demographics								
	**ANU-ADRI β [95%CI]**	**CAIDE β [95%CI]**	**CogDrisk β [95%CI]**	**LIBRA β [95%CI]**	**LIBRA2 β [95%CI]**	**UKBDRS β [95%CI]**	**UKBDRS-APOE β [95%CI]**	**LANCET β [95%CI]**
**Intercept**	24.75*** [24.64, 24.87]	24.65*** [24.53, 24.76]	24.85*** [24.72, 24.97]	24.90*** [24.79, 25.02]	24.88*** [24.76, 25.00]	24.57*** [24.45, 24.69]	24.62*** [24.48, 24.75]	24.75*** [24.63, 24.87]
**Time (per year)**	-0.29*** [-0.32, -0.26]	-0.26*** [-0.29, -0.23]	-0.25*** [-0.28, -0.22]	-0.28*** [-0.31, -0.25]	-0.27*** [-0.30, -0.24]	-0.25*** [-0.28, -0.22]	-0.23*** [-0.26, -0.19]	-0.27*** [-0.30, -0.24]
**Medium Risk (vs Low)**	-0.61*** [-0.81, -0.40]	-0.12 [-0.34, 0.10]	-0.63*** [-0.82, -0.44]	-0.76*** [-0.95, -0.56]	-0.64*** [-0.83, -0.44]	-0.04 [-0.27, 0.18]	-0.23* [-0.43, -0.02]	-0.35** [-0.55, -0.15]
**High Risk (vs Low)**	-0.81*** [-1.04, -0.59]	-0.75*** [-0.97, -0.54]	-1.06*** [-1.31, -0.82]	-1.29*** [-1.53, -1.05]	-1.22*** [-1.45, -0.99]	-0.43*** [-0.63, -0.22]	-0.36** [-0.58, -0.15]	-0.99*** [-1.21, -0.76]
**Time × Medium Risk**	0.02 [-0.04, 0.07]	-0.08** [-0.14, -0.02]	-0.07** [-0.12, -0.02]	-0.03 [-0.08, 0.02]	-0.05* [-0.10, -0.00]	-0.10** [-0.16, -0.04]	-0.07** [-0.12, -0.02]	-0.01 [-0.06, 0.05]
**Time × High Risk**	-0.03 [-0.08, 0.03]	-0.07* [-0.12, -0.01]	-0.10** [-0.16, -0.04]	-0.05 [-0.11, 0.01]	-0.05 [-0.11, 0.01]	-0.09** [-0.14, -0.04]	-0.15*** [-0.20, -0.09]	-0.10** [-0.16, -0.04]
**Var(Intercept)**	6.48	6.48	6.37	6.31	6.34	6.55	6.55	6.41
**Var(Time)**	0.31	0.31	0.30	0.31	0.30	0.31	0.30	0.30
**AIC**	45 526.68	45 526.54	45 470.67	45 441.11	45 454.66	45 554.95	45 544.60	45 490.15

Notes: All baseline participants (n=7221) and follow up participants (n=4716) are included; * p≤0.05; ** p≤0.01, *** p≤0.001)

Risk Scores Predicting Cognitive Decline by Age and Sex

Main Results

Supplementary Table 8) Mixed models with age and sex interaction terms, using IPW, random intercept, random slopes.

	ANU-ADRI β [95%CI]	CAIDE β [95%CI]	CogDrisk β [95%CI]	LIBRA β [95%CI]	LIBRA2 β [95%CI]	UKBDRS β [95%CI]	UKBDRS-APOE β [95%CI]	LANCET β [95%CI]
**Intercept**								
MOCA HUNT4 70+	24.89*** [24.75, 25.03]	24.91*** [24.77, 25.06]	24.84*** [24.70, 24.98]	24.80*** [24.66, 24.94]	24.80*** [24.65, 24.95]	24.97*** [24.84, 25.10]	24.97*** [24.84, 25.11]	24.87*** [24.73, 25.02]
Over 65 (=1)	-1.68*** [-1.90, -1.46]	-1.67*** [-1.89, -1.45]	-1.60*** [-1.82, -1.38]	-1.62*** [-1.84, -1.40]	-1.65*** [-1.87, -1.43]	-1.60*** [-1.80, -1.40]	-1.65*** [-1.85, -1.45]	-1.61*** [-1.83, -1.39]
Male (=1)	-0.91*** [-1.11, -0.71]	-0.77*** [-0.97, -0.57]	-0.88*** [-1.08, -0.68]	-0.86*** [-1.06, -0.66]	-0.84*** [-1.04, -0.64]	-0.93*** [-1.13, -0.73]	-0.91*** [-1.11, -0.71]	-0.90*** [-1.10, -0.70]
Male + over 65	0.37* [0.06, 0.68]	0.42** [0.11, 0.73]	0.39* [0.08, 0.70]	0.35* [0.04, 0.66]	0.44** [0.13, 0.75]	0.39** [0.10, 0.68]	0.42** [0.13, 0.71]	0.37* [0.06, 0.68]
Index (z)	-0.38*** [-0.52, -0.24]	-0.19** [-0.33, -0.05]	-0.41*** [-0.55, -0.27]	-0.49*** [-0.63, -0.35]	-0.47*** [-0.61, -0.33]	-0.14 [-0.30, 0.02]	-0.19** [-0.33, -0.05]	-0.35*** [-0.51, -0.19]
Over 65 × Index	-0.25* [-0.47, -0.03]	-0.10 [-0.32, 0.12]	-0.21 [-0.43, 0.01]	-0.31** [-0.53, -0.09]	-0.25* [-0.47, -0.03]	-0.29* [-0.53, -0.05]	-0.15 [-0.37, 0.07]	-0.19 [-0.43, 0.05]
Male × Index	-0.12 [-0.32, 0.08]	-0.06 [-0.26, 0.14]	-0.14 [-0.34, 0.06]	-0.08 [-0.28, 0.12]	-0.11 [-0.31, 0.09]	-0.16 [-0.40, 0.08]	-0.03 [-0.23, 0.17]	-0.05 [-0.27, 0.17]
Male + over 65 × Index	0.29 [-0.04, 0.62]	0.04 [-0.27, 0.35]	0.29 [-0.02, 0.60]	0.30 [0.01, 0.59]	0.32* [0.02, 0.62]	0.35* [0.01, 0.69]	0.15 [-0.17, 0.47]	0.10 [-0.24, 0.44]
**Slope**								
Time	-0.22*** [-0.26, -0.18]	-0.23*** [-0.27, -0.19]	-0.23*** [-0.27, -0.19]	-0.22*** [-0.26, -0.18]	-0.23*** [-0.27, -0.19]	-0.22*** [-0.26, -0.18]	-0.22*** [-0.26, -0.18]	-0.22*** [-0.26, -0.18]
Time + over 65	-0.20*** [-0.26, -0.14]	-0.22*** [-0.28, -0.16]	-0.21*** [-0.27, -0.15]	-0.21*** [-0.27, -0.15]	-0.20*** [-0.28, -0.12]	-0.20*** [-0.26, -0.14]	-0.21*** [-0.27, -0.15]	-0.20*** [-0.26, -0.14]
Time + male	0.02 [-0.04, 0.08]	0.04 [-0.02, 0.10]	0.03 [-0.03, 0.09]	0.03 [-0.03, 0.09]	0.03 [-0.03, 0.09]	0.02 [-0.04, 0.08]	0.02 [-0.04, 0.08]	0.02 [-0.04, 0.08]
Time + over 65 + male	-0.02 [-0.12, 0.08]	0.06 [-0.04, 0.16]	0.01 [-0.09, 0.11]	0.01 [-0.09, 0.11]	-0.00 [-0.10, 0.10]	0.02 [-0.08, 0.12]	0.04 [-0.06, 0.14]	0.00 [-0.10, 0.10]
Time × Index	-0.03 [-0.07, 0.01]	-0.04* [-0.08, -0.00]	-0.04 [-0.08, 0.00]	-0.03 [-0.07, 0.01]	-0.04 [-0.08, 0.00]	-0.07** [-0.11, -0.03]	-0.08*** [-0.12, -0.04]	-0.03 [-0.07, 0.01]
Time × over 65 × Index	0.05 [-0.01, 0.11]	-0.01 [-0.07, 0.05]	-0.00 [-0.06, 0.06]	0.00 [-0.06, 0.06]	0.03 [-0.03, 0.09]	0.05 [-0.03, 0.13]	0.02 [-0.04, 0.08]	0.03 [-0.05, 0.11]
Time × male × Index	0.03 [-0.03, 0.09]	0.03 [-0.03, 0.09]	0.04 [-0.02, 0.10]	0.02 [-0.04, 0.08]	0.04 [-0.02, 0.10]	0.05 [-0.01, 0.11]	0.03 [-0.03, 0.09]	0.00 [-0.06, 0.06]
Time × over 65 × male × Index	-0.15** [-0.25, -0.05]	-0.08 [-0.18, 0.02]	-0.09 [-0.19, 0.01]	-0.07 [-0.15, 0.01]	-0.13** [-0.23, -0.03]	-0.15** [-0.25, -0.05]	-0.06 [-0.16, 0.04]	-0.13* [-0.23, -0.03]
**Variance(time)**	0.48	0.34	1.16	0.60	0.35	0.29	0.39	0.24
**Variance(intercept)**	5.20	6.39	4.22	4.77	6.05	7.05	5.79	8.63
**AIC**	98778.15	98214.82	98227.41	98898.30	98475.59	97842.31	98512.29	96923.47

Notes: all baseline participants (n 7221) and follow up participants (4716) are included. Indices in this model are coded excluding age, educatior sex. (* p≤0.05; ** p≤0.01, *** p≤0.001)

Sensitivity Analsysis

Supplementary Table 9) Mixed models with age and sex interaction terms, using IPW, random intercept, random slopes. - No Inverse Probability Weights

	ANU-ADRI β [95%CI]	CAIDE β [95%CI]	CogDrisk β [95%CI]	LIBRA β [95%CI]	LIBRA2 β [95%CI]	UKBDRS β [95%CI]	UKBDRS-APOE β [95%CI]	LANCET β [95%CI]
**Intercept**								
MOCA HUNT4 70+	24.89*** [24.76, 25.03]	24.91*** [24.77, 25.06]	24.84*** [24.70, 24.98]	24.80*** [24.66, 24.94]	24.80*** [24.65, 24.95]	24.97*** [24.84, 25.10]	24.97*** [24.84, 25.11]	24.87*** [24.73, 25.02]
Over 65 (=1)	-1.69*** [-1.90, -1.48]	-1.69*** [-1.91, -1.46]	-1.62*** [-1.83, -1.41]	-1.64*** [-1.85, -1.42]	-1.67*** [-1.89, -1.45]	-1.62*** [-1.82, -1.41]	-1.66*** [-1.87, -1.46]	-1.63*** [-1.84, -1.41]
Male (=1)	-0.91*** [-1.11, -0.72]	-0.77*** [-0.97, -0.57]	-0.89*** [-1.09, -0.69]	-0.86*** [-1.06, -0.66]	-0.84*** [-1.04, -0.64]	-0.93*** [-1.13, -0.74]	-0.91*** [-1.11, -0.72]	-0.90*** [-1.11, -0.70]
Male + over 65	0.37* [0.06, 0.68]	0.42** [0.11, 0.74]	0.39* [0.09, 0.70]	0.36* [0.05, 0.66]	0.45** [0.14, 0.76]	0.39** [0.10, 0.69]	0.42** [0.13, 0.72]	0.38* [0.07, 0.68]
Index (z)	-0.38*** [-0.52, -0.24]	-0.19** [-0.33, -0.05]	-0.41*** [-0.55, -0.27]	-0.49*** [-0.62, -0.36]	-0.47*** [-0.61, -0.32]	-0.15 [-0.31, 0.01]	-0.19** [-0.33, -0.05]	-0.36*** [-0.51, -0.20]
Over 65 × Index	-0.26* [-0.48, -0.04]	-0.11 [-0.33, 0.11]	-0.22* [-0.43, -0.01]	-0.32** [-0.52, -0.11]	-0.26* [-0.49, -0.04]	-0.29* [-0.52, -0.05]	-0.15 [-0.37, 0.07]	-0.20 [-0.43, 0.03]
Male × Index	-0.12 [-0.33, 0.08]	-0.06 [-0.26, 0.15]	-0.13 [-0.33, 0.06]	-0.07 [-0.26, 0.12]	-0.11 [-0.31, 0.09]	-0.16 [-0.38, 0.07]	-0.03 [-0.24, 0.17]	-0.04 [-0.25, 0.17]
Male + over 65 × Index	0.29 [-0.03, 0.62]	0.05 [-0.27, 0.37]	0.29 [-0.02, 0.60]	0.30 [0.00, 0.59]	0.32* [0.00, 0.64]	0.35* [0.02, 0.68]	0.15 [-0.16, 0.47]	0.11 [-0.23, 0.45]
**Slope**								
Time	-0.21*** [-0.25, -0.17]	-0.21*** [-0.26, -0.17]	-0.22*** [-0.26, -0.17]	-0.21*** [-0.25, -0.17]	-0.22*** [-0.26, -0.18]	-0.21*** [-0.25, -0.17]	-0.20*** [-0.24, -0.17]	-0.21*** [-0.25, -0.17]
Time + over 65	-0.16*** [-0.22, -0.09]	-0.18*** [-0.25, -0.11]	-0.17*** [-0.24, -0.11]	-0.17*** [-0.24, -0.10]	-0.16*** [-0.23, -0.09]	-0.17*** [-0.23, -0.10]	-0.18*** [-0.24, -0.11]	-0.16*** [-0.22, -0.09]
Time + male	0.02 [-0.03, 0.08]	0.04 [-0.02, 0.10]	0.03 [-0.02, 0.09]	0.03 [-0.03, 0.09]	0.04 [-0.02, 0.10]	0.02 [-0.03, 0.08]	0.02 [-0.04, 0.07]	0.02 [-0.04, 0.08]
Time + over 65 + male	-0.02 [-0.12, 0.07]	0.04 [-0.05, 0.14]	0.00 [-0.09, 0.10]	0.00 [-0.09, 0.10]	-0.01 [-0.10, 0.09]	0.02 [-0.07, 0.10]	0.03 [-0.06, 0.12]	-0.01 [-0.10, 0.08]
Time × Index	-0.03 [-0.07, 0.01]	-0.04* [-0.08, 0.00]	-0.04* [-0.08, 0.00]	-0.03 [0.00, 0.00]	-0.04* [-0.08, 0.00]	-0.07** [-0.11, -0.02]	-0.08*** [-0.12, -0.04]	-0.04 [-0.08, 0.01]
Time × over 65 × Index	0.05 [-0.02, 0.12]	-0.01 [-0.07, 0.05]	0.00 [-0.06, 0.06]	0.01 [-0.05, 0.07]	0.03 [-0.04, 0.10]	0.05 [-0.02, 0.12]	0.02 [-0.05, 0.08]	0.05 [-0.02, 0.12]
Time × male × Index	0.03 [-0.03, 0.08]	0.03 [-0.03, 0.08]	0.04 [-0.01, 0.10]	0.03 [-0.03, 0.08]	0.04 [-0.01, 0.10]	0.05 [-0.02, 0.11]	0.02 [-0.03, 0.08]	0.01 [-0.05, 0.07]
Time × over 65 × male × Index	0.29 [-0.03, 0.62]	0.05 [-0.27, 0.37]	0.29 [-0.02, 0.60]	0.30 [0.00, 0.59]	0.32* [0.00, 0.64]	0.35* [0.02, 0.68]	0.15 [-0.16, 0.47]	0.11 [-0.23, 0.45]
**Variance(time)**	0.24	0.24	0.23	0.23	0.23	0.24	0.23	0.24
**Variance(intercept)**	6.87	7.02	6.80	6.66	6.76	7.04	7.03	6.91
**AIC**	58 601.84	58 711.13	58 564.26	58 472.09	58 533.53	58 702.32	58 698.62	58 629.42

Notes: all baseline participants (n 7221) and follow up participants (4716) are included. Indices in this model are coded excluding age, education and sex. (* p≤0.05; ** p≤0.01, *** p≤0.001).

Supplementary Table 10) Mixed models with age and sex interaction terms, using IPW, random intercept, random slopes. - Complete Cases Only

	ANU-ADRI β [95%CI]	CAIDE β [95%CI]	CogDrisk β [95%CI]	LIBRA β [95%CI]	LIBRA2 β [95%CI]	UKBDRS β [95%CI]	UKBDRS-APOE β [95%CI]	LANCET β [95%CI]
**Intercept**								
MOCA HUNT4 70+	25.12*** [24.96, 25.27]	25.17*** [25.00, 25.33]	25.06*** [24.90, 25.22]	24.997*** [24.83, 25.16]	25.01*** [24.84, 25.18]	25.24*** [25.09, 25.39]	25.25*** [25.10, 25.39]	25.07*** [24.91, 25.24]
Over 65 (=1)	-1.09*** [-1.35, -0.84]	-1.09*** [-1.37, -0.82]	-1.04*** [-1.30, -0.78]	-1.06*** [-1.32, -0.80]	-1.08*** [-1.35, -0.81]	-1.05*** [-1.29, -0.81]	-1.09*** [-1.34, -0.85]	-0.97*** [-1.23, -0.72]
Male (=1)	-0.91*** [-1.14, -0.69]	-0.75*** [-0.98, -0.52]	-0.86*** [-1.10, -0.63]	-0.83*** [-1.06, -0.59]	-0.81*** [-1.05, -0.58]	-0.92*** [-1.14, -0.70]	-0.91*** [-1.13, -0.69]	-0.83*** [-1.07, -0.59]
Male + over 65	0.28 [-0.09, 0.66]	0.27 [-0.10, 0.64]	0.28 [-0.09, 0.65]	0.24 [-0.13, 0.62]	0.33 [0.00, 0.64]	0.29 [-0.07, 0.64]	0.31 [-0.04, 0.66]	0.19 [-0.19, 0.56]
Index (z)	-0.45*** [-0.61, -0.29]	-0.21** [-0.37, -0.05]	-0.46*** [-0.62, -0.31]	-0.56*** [-0.71, -0.41]	-0.53*** [-0.69, -0.36]	-0.14 [-0.32, 0.05]	-0.14 [-0.30, 0.02]	-0.49*** [-0.66, -0.32]
Over 65 × Index	-0.10 [-0.37, 0.16]	-0.09 [-0.35, 0.17]	-0.10 [-0.35, 0.15]	-0.18 [-0.42, 0.06]	-0.16 [-0.43, 0.10]	-0.27 [-0.57, 0.03]	-0.20 [-0.47, 0.08]	0.07 [-0.21, 0.35]
Male × Index	-0.10 [-0.34, 0.13]	-0.05 [-0.28, 0.19]	-0.07 [-0.29, 0.16]	-0.00 [-0.22, 0.22]	-0.04 [-0.28, 0.19]	-0.13 [-0.40, 0.15]	-0.09 [-0.32, 0.15]	0.11 [-0.13, 0.36]

Male + over 65 × Index	0.28 [-0.11, 0.66]	0.05 [-0.33, 0.43]	0.21 [-0.15, 0.58]	0.19 [-0.16, 0.54]	0.27 [-0.10, 0.64]	0.46* [0.03, 0.88]	0.31 [-0.07, 0.70]	-0.08 [-0.49, 0.32]
**Slope**								
Time	-0.22*** [-0.26, -0.18]	-0.23*** [-0.27, -0.19]	-0.23*** [-0.27, -0.19]	-0.23*** [-0.27, -0.18]	-0.23*** [-0.28, -0.19]	-0.23*** [-0.26, -0.19]	-0.22*** [-0.26, -0.19]	-0.23*** [-0.27, -0.18]
Time + over 65	-0.20*** [-0.27, -0.14]	-0.22*** [-0.29, -0.15]	-0.22*** [-0.28, -0.15]	-0.21*** [-0.28, -0.15]	-0.20*** [-0.27, -0.13]	-0.21*** [-0.27, -0.14]	-0.22*** [-0.28, -0.16]	-0.21*** [-0.27, -0.14]
Time + male	0.02 [-0.03, 0.08]	0.04 [-0.02, 0.10]	0.03 [-0.03, 0.09]	0.03 [-0.03, 0.09]	0.04 [-0.02, 0.10]	0.02 [-0.03, 0.08]	0.02 [-0.04, 0.07]	0.02 [-0.04, 0.08]
Time + over 65 + male	-0.02 [-0.11, 0.08]	0.05 [-0.04, 0.15]	0.01 [-0.08, 0.11]	0.01 [-0.09, 0.11]	0.00 [-0.09, 0.10]	0.02 [-0.07, 0.11]	0.04 [-0.05, 0.13]	0.00 [-0.09, 0.10]
Time × Index	-0.03 [-0.07, 0.01]	-0.04 [-0.08, -0.00]	-0.04 [-0.03, -0.05]	-0.03 [-0.06, 0.01]	-0.04 [-0.03, -0.05]	-0.07** [-0.11, -0.02]	-0.08*** [-0.12, -0.04]	-0.03 [-0.07, 0.02]
Time × over 65 × Index	0.04 [-0.03, 0.11]	-0.01 [-0.07, 0.05]	-0.01 [-0.07, 0.06]	-0.00 [-0.06, 0.06]	0.02 [-0.05, 0.09]	0.05 [-0.02, 0.12]	0.02 [-0.05, 0.09]	0.03 [-0.05, 0.10]
Time × male × Index	0.02 [-0.03, 0.08]	0.03 [-0.03, 0.08]	0.04 [-0.02, 0.10]	0.02 [-0.03, 0.08]	0.04 [-0.02, 0.10]	0.04 [-0.02, 0.11]	0.03 [-0.03, 0.09]	-0.00 [-0.06, 0.06]
Time × over 65 × male × Index	-0.14** [-0.24, -0.04]	-0.08 [-0.17, 0.02]	-0.08 [-0.17, 0.01]	-0.07 [-0.16, 0.02]	-0.12** [-0.22, -0.03]	-0.14** [-0.25, -0.04]	-0.06 [-0.16, 0.03]	-0.12* [-0.22, -0.02]
**Variance(time)**	0.28	0.27	0.27	0.27	0.27	0.28	0.27	0.27
**Variance(intercept)**	5.85	5.98	5.78	5.66	5.75	6.02	6.00	5.89
**AIC**	45 116.28	45 188.78	45 084.98	45 015.59	45 059.98	45 197.96	45 182.49	45 141.06

Notes: all baseline participants (n 7221) and follow up participants (4716) are included. Indices in this model are coded excluding age, education and sex. (* p≤0.05; ** p≤0.01, *** p≤0.001).

Predicting Cognitive Decline as a Binary Outcome

Sensitivity Analysis

Supplementary Table 11) Logistic regression of continuous risk score predicting binary cognitive change (2SD).

With Demographics (Change of ≥ 6(2SD) MoCA points n = 450; No Change = 4261)

Risk Score	Coefficient	95% CI	AIC	AUC	AUC 95% CI
**Age**	0.11***	0.09,0.13	4629.55	0.65	0.62, 0.67
**Education**	-0.34**	-0.56, -0.12			
**ANU-ADRI**	0.82***	0.6, 0.97	4668.13	0.63	0.61, 0.66
**CAIDE**	0.24***	0.14, 0.34	4893.68	0.57***	0.54, 0.59
**CogDrisk**	0.79***	0.64, 0.93	4684.89	0.64	0.61, 0.67
**LIBRA**	0.81***	0.67, 0.96	4663.07	0.64	0.62, 0.67
**LIBRA2 (uLIBRA)**	0.42***	0.31, 0.54	4833.07	0.59**	0.56, 0.62
**UKBDRS**	0.90***	0.75, 1.05	4633.99	0.65	0.62, 0.67
**UKBDRS-APOE**	0.90***	0.75, 1.05	4629.85	0.66	0.63, 0.68
**Lancet**	0.85***	0.71, 1.00	4649.10	0.63	0.60, 0.66

No Demographics Included

Risk Score	Coefficient	95% CI	AIC	AUC	AUC 95% CI
**ANU-ADRI**	0.17**	0.06, 0.28	4913.72	0.54	0.52, 0.57
**CAIDE**	0.14**	0.04, 0.24	4917.76	0.54	0.51, 0.56
**CogDrisk**	0.23***	0.13, 0.33	4899.41	0.56	0.53, 0.58
**LIBRA**	0.24***	0.14, 0.35	4894.20	0.56	0.53, 0.59

**LIBRA2**	0.22***	0.12, 0.32	4901.00	0.55	0.53, 0.58
**UKBDRS**	0.22***	0.12, 0.31	4901.47	0.57	0.54, 0.60
**UKBDRS-APOE**	0.22***	0.12, 0.31	4900.64	0.57	0.55, 0.60
**Lancet**	0.21***	0.11, 0.32	4903.47	0.55	0.52, 0.58

Notes: Significance in AUC differences is tested using DeLong tests; * p≤0.05; ** p≤0.01, *** p≤0.001

Supplementary Table 12) Logistic regression of continuous risk score predicting binary cognitive change (3SD).

With Demographics (Change of ≥ 9(3SD) MoCA points n = 118; No Change = 4593)

**Risk Score**	**Coefficient**	**95% CI**	**AIC**	**AUC**	**AUC 95% CI**
**Age**	0.14***	0.11, 0.18	1792.47	0.71	0.66, 0.76
**Education**	-0.60**	-1.05, -0.15			
**ANU-ADRI**	1.03***	0.79, 1.26	1838.68	0.69	0.64, 0.74
**CAIDE**	0.30***	0.12, 0.47	1959.80	0.58***	0.53, 0.63
**CogDrisk**	0.94***	0.69, 1.19	1867.75	0.68	0.63, 0.73
**LIBRA**	1.04***	0.80, 1.28	1829.23	0.69	0.64, 0.74
**LIBRA2 (uLIBRA)**	0.38***	0.17, 0.59	1959.78	0.59***	0.54, 0.63
**UKBDRS**	1.15***	0.90, 1.40	1818.13	0.70	0.65, 0.76
**UKBDRS-APOE**	1.19***	0.93, 1.44	1805.84	0.71	0.66, 0.76
**Lancet**	1.15***	0.91, 1.38	1818.90	0.70	0.64, 0.76

No Demographics Included

**Risk Score**	**Coefficient**	**95% CI**	**AIC**	**AUC**	**AUC 95% CI**
**ANU-ADRI**	-0.03	-0.21, 0.15	1980.20	0.50	0.45, 0.54
**CAIDE**	0.14	-0.06, 0.33	1976.54	0.54	0.49, 0.59
**CogDrisk**	0.05	-0.14, 0.23	1979.90	0.52	0.46, 0.57
**LIBRA**	0.14	-0.05, 0.33	1976.46	0.53*	0.48, 0.58
**LIBRA2**	0.06	-0.15, 0.26	1979.76	0.51	0.46, 0.56
**UKBDRS**	0.19*	0.04, 0.35	1977.00	0.59*	0.54, 0.64
**UKBDRS-APOE**	0.28**	0.10, 0.45	1968.18	0.60*	0.55, 0.65
**Lancet**	0.14	-0.07, 0.35	1978.69	0.53	0.48, 0.59

Notes: Significance in AUC differences is tested using DeLong tests; * p≤0.05; ** p≤0.01, *** p≤0.001

References

1. Næss M, Kvaløy K, Sørgjerd EP, Sætermo KS, Norøy L, et al. Data Resource Profile: The HUNT Biobank. International Journal of Epidemiology. 2024;53(3).
2. Weir CB JA. BMI Classification Percentile And Cut Off Points. National Library of Medicine: StatPearls Treasure Island; 2023. Available from: https://www.ncbi.nlm.nih.gov/books/NBK541070/.
3. Hinz A, Brähler E. Normative values for the Hospital Anxiety and Depression Scale (HADS) in the general German population. Journal of Psychosomatic Research. 2011;71(2):74-8.
4. Akbar DH. Sub-optimal postprandial blood glucose level in diabetics attending the outpatient clinic of a University Hospital. Saudi Med J. 2003;24(10):1109-12.
5. Kivipelto M, Ngandu T, Laatikainen T, Winblad B, Soininen H, et al. Risk score for the prediction of dementia risk in 20 years among middle aged people: a longitudinal, population-based study. Lancet Neurology. 2006;5(9):735--41.
6. Skjellegrind HK, Thingstad P, Gjøra L, Kolberg M, Kjelvik G, et al. Cohort profile update: HUNT4 70+. medRxiv. 2025:2025.06.24.25330190.
